# Cycloartane Saponins from *Astragalus glycyphyllos* and Their In Vitro Neuroprotective, Antioxidant, and hMAO-B-Inhibiting Effects

**DOI:** 10.3390/metabo13070857

**Published:** 2023-07-19

**Authors:** Ivan Stambolov, Aleksandar Shkondrov, Olaf Kunert, Franz Bucar, Magdalena Kondeva-Burdina, Ilina Krasteva

**Affiliations:** 1 Department of Pharmacognosy, Faculty of Pharmacy, Medical University of Sofia, 2 Dunav St., 1000 Sofia, Bulgaria; istambolov@pharmfac.mu-sofia.bg (I.S.); shkondrov@pharmfac.mu-sofia.bg (A.S.); 2 Department of Pharmaceutical Chemistry, Institute of Pharmaceutical Sciences, University of Graz, Universitätsplatz 1, A-8010 Graz, Austria; olaf.kunert@uni-graz.at; 3 Department of Pharmacognosy, Institute of Pharmaceutical Sciences, University of Graz, Beethovenstrasse 8, A-8010 Graz, Austria; franz.bucar@uni-graz.at; 4 Laboratory of Drug Metabolism and Drug Toxicity, Department of Pharmacology, Pharmacotherapy and Toxicology, Faculty of Pharmacy, Medical University of Sofia, 2 Dunav St., 1000 Sofia, Bulgaria; mkondeva@pharmfac.mu-sofia.bg

**Keywords:** cycloartane saponins, isolation, structural elucidation, neuroprotection, antioxidant activity, hMAO-B-inhibition, brain synaptosomes, brain mitochondria, brain microsomes

## Abstract

*Astragalus glycyphyllos* (Fabaceae) is used in the traditional medicine of many countries against hepatic and cardiac disorders. The plant contains mainly flavonoids and saponins. From a defatted methanol extract from its overground parts, a new triterpenoid saponin, 3-*O*-[*α*-L-rhamnopyranosyl-(1→2)]-*β*-D-xylopyranosyl]-24-*O*-*α*-L-arabinopyranosyl-3*β*,6*α*,16*β*,24(*R*),25-pentahydroxy-20*R*-cycloartane, together with the rare saponin astrachrysoside A, were isolated using various chromatography methods. The compounds were identified via extensive high resolution electrospray ionisation mass spectrometry (HRESIMS) and NMR analyses. Both saponins were examined for their possible antioxidant and neuroprotective activity in three different in vitro models. Rat brain synaptosomes, mitochondria, and microsomes were isolated via centrifugation using Percoll gradient. They were treated with the compounds in three different concentrations alone, and in combination with 6-hydroxydopamine or *tert*-butyl hydroperoxide as toxic agents. It was found that the compounds had statistically significant dose-dependent in vitro protective activity on the sub-cellular fractions. The compounds exhibited a weak inhibitory effect on the enzyme activity of human recombinant monoamine oxidase type B (hMAO-B), compared to selegiline.

## 1. Introduction

*Astragalus glycyphyllos* L., Fabaceae (Liquorice Milk-Vetch) is a perennial herbaceous plant with deep roots, distributed in the mountainous regions of Bulgaria on stony places in forest glades, forests, and scrub, up to 1750 m above sea level [1,2]. The species has been widely used in the folk medicine of the country as an anti-inflammatory, antihypertensive, and diuretic, etc. Its aerial parts can be used as an infusion for heart failure, kidney inflammation, and calculus, and as an adjuvant treatment for cancer diseases, tachycardia, and increased blood pressure, etc. [3]. Research on *A. glycyphyllos* has focused on its biologically active secondary metabolites such as saponins and flavonoids [4]. A phytochemical investigation of the overground parts of the species was initiated in Bulgaria 35 years ago and led to the isolation of some sapogenins (soyasapogenol B and 3*β*,22*β*,24-trihydroxyolean-12-en-19-one) after acid hydrolysis [5,6]. In later research, the saponins askendoside C and F were isolated from the roots of the plant [7]. Recently, from its aerial parts, an epoxycycloartane saponin 17(*R*),20(*R*)-3*β*,6*α*,16*β*-trihydroxycycloartanyl-23-carboxilic acid 16-lactone 3-*O*-*β*-D-glucopyranoside [8] was isolated. 

A defatted extract obtained from the aerial parts of the species had in vivo antioxidant and hepatoprotective effects on Wistar rats with carbon tetrachloride-induced hepatotoxicity. The effects of the extract, containing mainly flavonoids and saponins, were commensurable to those of the classical hepatoprotector silymarin [9]. A purified saponin mixture (PSM) resulting from the same extract displayed antiproliferative and cytotoxic effects in vitro/in vivo on Graffi myeloid tumour cells and Graffi-tumour-bearing hamsters. Per oral treatment with PSM extended survival and reduced tumour growth. A statistically significant decrease in the proliferation and viability of the tumour cells was observed after this administration. Concentration- and time-dependent effects were proved. The antiproliferative effects were related to the induction of apoptosis by the saponins, which was demonstrated using fluorescence microscopy [10]. Several compounds obtained from the aerial parts, including the epoxycycloartane saponin, exhibited neuroprotective activity on 6-hydroxydopamine-induced neurotoxicity in isolated rat brain synaptosomes. The same saponin also displayed statistically significant hMAO-B-enzyme-inhibiting activity compared to selegiline [8]. Based on these data, the saponins from this species could be considered as perspective, possessing valuable pharmacological activities.

Neurodegenerative diseases, especially Parkinson’s disease, are a major health concern. For the last two decades, they have established a new group of socially important ailments, which have a major impact on our modern life [11]. In pharmacological research, in vitro systems play an important role as the initial data collection tool. Their role in evaluating the ability of a compound to influence the mechanism of some disorders is invaluable. In vitro methods are fast and more acceptable than in vivo ones, especially in ethical terms. Unfortunately, the extrapolation of these results to a living organism is not always possible or adequate. For some pathogenetic mechanisms, their underlying factors are known. This could be reproduced in an in vitro model to accumulate results commensurable to those processes that are responsible for this effect in vivo [12,13]. Oxidative stress is considered to be the leading risk factor resulting in neurodegeneration. Thus, neuroprotection is in direct connection with antioxidant activity [11]. Sub-cellular fractions (synaptosomes, mitochondria, and microsomes) are a convenient object for the investigation of neuronal damage and its mechanisms, as included in [14]. The availability of specific enzymes (incl. human recombinant ones) capable of metabolizing neurotransmitters is another advantage of today’s in vitro methods. It is known that human monoamine oxidase type B is responsible for metabolizing dopamine, so inhibiting it is a way of increasing the depleted dopamine in conditions such as Parkinson’s [15].

In this continuation of our research, the aim was to isolate more polar and previously unidentified saponins from *Astragalus glycyphyllos* and to evaluate their possible antioxidant, neuroprotective, and hMAO-B-inhibiting effects in in vitro models.

## 2. Materials and Methods

### 2.1. General

AUTOPOL VI (Rudolph Research Analytical) was used to measure optical rotation. NMR spectra were recorded with a 700 MHz Bruker Avance II NMR spectrometer (Bruker, Rheinstetten, Germany; software MNova, Mestrelab Research, Santiago de Compostela, Spain) equipped with a cryo-probe. For both compounds, **S1** and **S2,** a data set consisting of 1D proton and carbon experiments and 2D COSY, HSQC, HMBC, and ROESY experiments was acquired at 298 K in methanol-*d*_4_; in addition, a second data set for S2 was recorded at the same temperature in pyridine-*d*_5_.

A UHPLC system (Dionex UltiMate 3000 RSLC, ThermoFisher Scientific, Bremen, Germany) connected to a Q Exactive Plus Orbitrap mass spectrometer with a heated electrospray ionisation (HESI) ion source (ThermoFisher Scientific, Bremen, Germany) was used. The parameters of the full scan MS were in the resolution of 70,000 (@200 *m*/*z*), 3e^6^AGC target, 100 ms max IT, and 250 to 1700 *m*/*z* scan range. The ion source operated at −2.5 or +3.5 kV and 320 °C (capillary and probe), 38 arbitrary units (a.u., as by the software Extactive Tune, ThermoFisher Scientific, Bremen, Germany) of sheath gas, and 12 a.u. of auxiliary gas (both N_2_); an S-Lens RF level 50.0. A Kromasil C_18_ column (1.9 μm, 2.1 × 50 mm, Akzo Nobel, Bohus, Sweden) was used, maintained at 40 °C, and eluted with H_2_O + 0.1% HCOOH (A) and MeCN + 0.1% HCOOH (B) (0.3 mL/min). The gradient program was for 0.5 min 10% B, for 7 min increasing to 30% B, for 1.5 min 30% B (isocratic), for 3.5 min increasing to 95% B, for 2 min with 95% B (isocratic), and for 0.1 min decreasing to 10% B. Diaion HP-20 resin was purchased from Supelco (Bellefonte, PA, USA). Silica gel cartridges (FlashPure*^®^* 40 µm, irregular, Buchi, Flawil, Switzerland) were used on a Reveleris X2 flash chromatograph (Buchi, Flawil, Switzerland). Sephadex LH-20 was obtained from Supelco (Bellefonte, PA, USA). TLC was performed on Kieselgel F_254_ sheets (Merck, Darmstadt, Germany), eluted with EtOAc/HCOOH/AcOH/H_2_O (32/3/2/6). The saponins were visualised using p-anisaldehyde/conc. H_2_SO_4_ spraying and heating for 10 min at 104 °C were performed.

The determination of the absolute configuration of the monosaccharides forming the sugar chains was performed using a known GC-MS method [16]. Briefly, after acid hydrolysis, (2R)-2-butyl glycosides were prepared. Each compound (2 mg) was heated with 5.5 mL of H_2_O, 3.5 mL of conc. AcOH, and 1 mL of conc. HCl on a water bath at 100 °C under a reflux condenser for 2 h. The hydrolysate was extracted with EtOAc and the aqueous residue was evaporated to dryness. To the residue, 0.45 mL of (2R)-2-BuOH and 0.1 mL of conc. HCl were added and heated at 100 °C for 15 h. The mixture was evaporated under N_2_, and then 100 μL of Sigma-Sil-A (Sigma-Aldrich, Schnelldorf, Germany) was added to prepare TMS derivatives. The standard compounds (L-rhamnose, D-xylose, and L-arabinose, Sigma Aldrich, Schnelldorf, Germany) were treated in the same manner. The silated derivatives of the (2R)-2-butyl glycosides formed were analysed using GC-MS. For the GC-MS analysis, an Exactive Orbitrap GC-MS system (ThermoFisher Scientific, Bremen, Germany) was used. It was operated at 70 eV, with a 230 °C ion source, 280 °C interface, 270 °C injector temperature, and 1 μL injection volume (split, 20:1 ratio). A capillary column (fused silica, 5% phenyl/95% methyl polysiloxane, HP-5MS 30 m × 250 μm × 0.25 μm, Agilent, Santa Clara, CA, USA) was used. The temperature program was initially 100 °C at 3 °C/min to 270 °C. Helium 5.0 (1.5 mL/min) was the carrier gas. The data (50–450 u) were collected with Xcalibur v. 4 (ThermoFisher Scientific, Bremen, Germany).

All other chemicals and solvents were obtained from Sigma-Aldrich (Schnelldorf, Germany).

### 2.2. Extraction and Isolation of Compounds from Plant Material

The *A. glycyphyllos* overground parts were harvested in July 2020 from Vitosha Mt., Bulgaria. Prof. D. Pavlova identified the plant, and a specimen is kept in the Herbarium of the Faculty of Biology, Sofia University (S0 107 613). The air-dried plant material (200 g) was powdered (3 mm) and then extracted with dichloromethane (6 *×* 2 L) using percolation to remove the lipophilic constituents. The defatted plant substance was then aired and exhaustively extracted with 80% MeOH (24 × 3 L) using percolation. The obtained extract was filtered, concentrated under vacuum, and then lyophilized to produce a dry extract (42 g). The extract was separated over a Diaion HP-20 (4.7 × 45) column, eluting with H_2_O: MeOH (0→100%). Seven main fractions were collected (I-VII). After the TLC analysis, fractions VI and VII were found to be rich in saponins. Fraction VI was chromatographed with CH_2_Cl_2_:MeOH:H_2_O (step gradient 8:2:0.2→7:3:0.3) on a silica gel cartridge using flash chromatography to obtain 21 subfractions. Subfraction 13, which contained a main compound (TLC), was further separated with CH_2_Cl_2_:MeOH:H_2_O (step gradient 9:1:0→5:6:1) on a silica gel cartridge using flash chromatography, affording compound **S1**. The saponin was further purified over Sephadex LH-20 (eluent MeOH) to obtain 33 mg of it. Fraction VII, containing another saponin (TLC analysis), was separated two subsequent times using flash chromatography on a silica gel cartridge with CH_2_Cl_2_:MeOH:H_2_O (step gradient 8:2:0.1→5:5:0.5), to obtain compound **S2**. The final purification of the saponin over Sephadex LH-20 (eluent MeOH) provided 20 mg of S2. 

*3-O-[α-L-rhamnopyranosyl-(1→2)]-β-D-xylopyranosyl]-24-O-α-L-arabinopyranosyl-3β,6α,16β,24(R),25-pentahydroxy-20R-cycloartane* (**S1**): a white amorphous powder (MeOH); C_46_H_78_O_17_; ^1^H NMR (methanol-*d*_4_, 700 MHz), see Table 1, Figures S1–S3; ^13^C NMR (methanol-*d*_4_, 175 MHz), see Table 1, Figure S4; HREIMS, *m*/*z* 901.5186 [M − H]^−^ (calcd. for C_46_H_77_O_17_, 901.5160), *m*/*z* 947.5242 [M + HCOO]^−^ (calcd. for C_47_H_79_O_19_, 947.5215), Figure S28; m/z 903.5323 [M + H]^+^ (calcd. for C_46_H_79_O_17_, 903.5317), *m*/*z* 925.5142 [M + Na]^+^ (calcd. for C_46_H_78_O_17_Na, 925.5136), Figure S29; [α]^D^_20_ = −1.0546 (c 0.1, MeOH).

### 2.3. In Vitro Pharmacological Evaluation

Twenty Wistar rats (male, 200–250 g) were purchased from the National Breeding Centre of the Bulgarian Academy of Sciences in Slivnitsa, Bulgaria. They were kept in Plexiglas cells (3 in each) and a seven-day acclimatization period was allowed before the commencement of the experiment. A veterinary physician monitored the animals’ health daily. The rats were given standardized pellets chew and drinking water ad libitum. The husbandry conditions in the Vivarium of the Faculty of Pharmacy at the Medical University of Sofia were checked by the Bulgarian Food Safety Agency at the Bulgarian Ministry of Foods and Agriculture. The experiment was allowed by permission № 200/2021 from the same agency. The University Ethical Commission (KENIMUS) gave ethical clearance for the experiment with the protocol 7338/11.2021. 

For the isolation of the sub-cellular structures, the rats were decapitated, the cranial cavities were opened, and the brains were removed and stored over ice. The organs were pooled and homogenised using a Teflon pestle with the appropriate buffers, as described in the procedures below. 

The synaptosomes and brain mitochondria were isolated using multiple differential centrifugations with the methods of [17,18]. Two buffers—A (HEPES 5 mM and Sucrose 0.32 M) and B (NaCl 290 mM, MgCl_2_.2H_2_O 0.95 mM, KCl 10 mM, CaCl_2_.2H_2_O 2.4 mM, NaH_2_PO_4_ 2.1 mM, HEPES 44 mM, and D-Glucose 13 mM)—were prepared. Buffer A was used to prepare the brain homogenate. First, the homogenate was centrifuged twice at 1000× *g* for 10 min at 4 °C, after which, the supernatants of the two centrifuges were combined and centrifuged three times at 10,000× *g* for 20 min at 4 °C. The isolation of the synaptosomes and mitochondria was accomplished using Percoll. First, a 90% Percoll stock solution was prepared and, after that, 16% and 10% solutions were prepared. Amounts of 4 mL of 16% and 10% Percoll were carefully applied in layers. At the end, 4 mL of 7.5% Percoll was added to the precipitate from the last centrifugation. The tubes were centrifuged at 15,000× *g* for 20 min at 4 °C. After this centrifugation, three layers were formed: the lower, which contained mitochondria; the medium (between 16% and 10% Percoll), containing synaptosomes; and the top, lipids. Using a Pasteur pipette, we removed the middle and bottom layers. Each was centrifuged at 10,000× *g* for 20 min at 4 °C with buffer B. The resulting synaptosomes and mitochondria were diluted with buffer B to a protein content of 0.1 mg/mL. After incubation with the test substances (saponins) and 6-OHDA, the synaptosomes were centrifuged three times at 15,000× *g* for 1 min. The synaptosomal viability was determined using an MTT test with the method of [19]. After incubation with the substances (saponins), the synaptosomes were centrifuged at 400× *g* for 3 min. The precipitate was treated with 5% trichloroacetic acid, vortexed, and left on ice for 10 min, then centrifuged at 8000× *g* for 10 min. The supernatant was frozen at −20 °C. Immediately prior to their determination, each sample was neutralized with 5 M NaOH. The GSH levels were determined using Elmman’s reagent (DTNB) spectrophotometrically at 412 nm with methods of [20,21]. For the isolation of the brain microsomes, the brain was homogenized in 9 volumes of 0.1 M Tris buffer containing 0.1 mM Dithiothreitol, 0.1 mM Phenylmethylsulfonyl fluoride, 0.2 mM EDTA, 1.15% KCl, and 20% (*v*/*v*) glycerol (pH 7.4). The resulting homogenate was centrifuged twice at 17,000× *g* for 30 min. The supernatants from the two centrifuges were combined and centrifuged twice at 100,000× *g* for 1 h. The pellet was frozen in 0.1 M Tris buffer at −20 °C [22]. The determination of MDA was performed spectrophotometrically at a wavelength of 535 nm using methods of [21,23]. A molar extinction coefficient of 1.56 × 10^5^ M^−1^ cm^−1^ was used for the calculation [24].

The examined compounds were tested for their possible inhibitory activity on the hMAO-B enzyme (commercially available) using the Amplex UltraRed reagent fluorometric method [25] with small modifications [15].

Salts to prepare the buffer, 2,2′-dinitro-5,5′-dithiodibenzoic acid, reduced glutathione (GSH), oxidized glutathione (GSSG), human recombinant MAO type B enzyme, tyramine HCl, and horseradish peroxidase, as well as other reagents, were used for the pharmacological study and obtained from Sigma-Aldrich (Schnelldorf, Germany). The Amplex UltraRed kit was from Invitrogen (Thermo Scientific, Karlsruhe, Germany)

The statistical analysis of the results was performed using the statistical programme “Medcalc” v. 18 (MedCalc Software Ltd., Ostend, Belgium). Each experiment was performed in triplicate, and the values are represented as the mean of three (*n* = 3). A Mann–Whitney non-parametric test was used to examine the statistical significance of the results. When *p* < 0.05; *p* < 0.01; or *p* < 0.001, the differences were accepted as significant.

## 3. Results

### 3.1. Identification of the Compounds

Compound **S1** was isolated as a white amorphous powder (33 mg). In the negative HRESIMS spectrum (Figures S28 and S29) of the compound, an ion [M − H]^−^ at *m*/*z* 901.5187 was observed, corresponding to the molecular formula C_46_H_78_O_17_. The ^1^H and ^13^C NMR (Table 1, Table S1, Figures S1–S10) and HSQC spectrum pointed to a triterpene of the cycloartane-type with three sugars. The number of methine groups attached to the oxygen atoms (from –OH) was higher than expected for the three sugars, indicating the presence of at least five oxygenated positions in the aglycone. The complete resonance assignments (Table 1) led to 3,6,16,20,24,25-hexahydroxycycloartane as the aglycone. A list of all the essential HMBC correlations and ROESY cross-peaks are provided in the supporting information (Table S1). From the ROESY cross-peaks, it was deduced that H-3, H-5, H-30, H-16, and H-17 were on one face of the molecule, while H-6, H-8, H-18, H-19, and H-18 were on the opposite. The positions C-3, C-6, C-16, C-24, and C-25 were oxidized. Hence, the aglycone of compound **S1** was assigned as 3*β*,6*α*,16*β*,24,25-hexahydroxycycloartane. The aglycone was bound to three sugar moieties. After the complete resonance assignments, an analysis of the coupling patterns, and a comparison of their carbon resonances with the literature values, the three sugars were identified as *β*-xylose, *α*-rhamnose, and *α*-arabinose. Two of those, the xylose and the rhamnose, had identical resonance values and HMBC correlations as observed for astrachrysoside A (**S2**) in methanol-*d*_4_ (Table 1 and Table S2); therefore, both S1 and S2 had the same saccharide structure attached to C-3. A more detailed discussion was conducted for compound **S2** (see below). The remaining sugar moiety, an *α*-arabinose, was attached to the C-24 position of the aglycone according to the observed HMBC correlation for its anomeric proton. 

**Figure 1:**
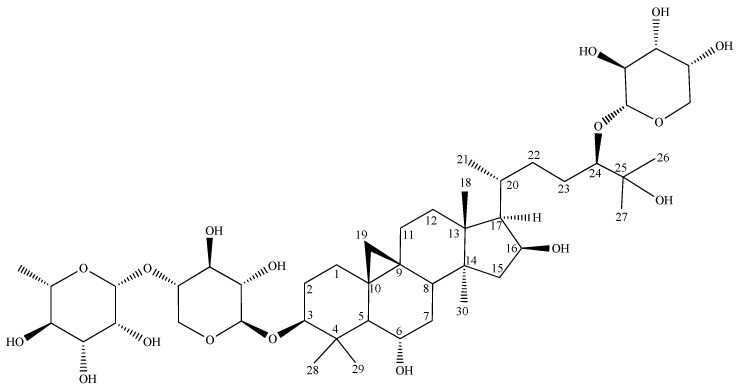
Structure of S1.

NMR data from various *Astragalus* species were available for the same type of glycosylated side chain with a different configuration at the chiral carbon C-24, e.g., with a *24S*-configuration from *A. brachypterus* [25] and a 24R-configuration from *A. stereocalyx* [26]. A comparison of the ^13^C-NMR shift values (Table 2) clearly indicated that, in compound **S1,** the position C-24 had an R-configuration. Additionally, a GC-MS analysis of the sugars after the preparation of the (2R)-butyl glycosides was performed to elucidate their absolute configuration. After the GC-MS analysis of the sililated (*2R*)-butylglycosides, it was found that S1 gave peaks at t_R_ = 24.97, 26.68 (L-Rha); 29.81, 31.42 (D-Xyl); and 24.62, 26.68 min (L-Ara), respectively (Figure S32) [16]. On basis of these spectroscopic data and the result of the sugar hydrolysis, the structure of S1 was established as 3-O-[*α*-L-rhamnopyranosyl-(1→2)]-*β*-D-xylopyranosyl]-24-O-*α*-L-arabinosyl-3*β*,6*α*,16*β*,24,25-pentahydroxy-20*R*,24*R*-cycloartane (Figure 1). To our knowledge, this is the first report on this compound.

**Figure 2:**
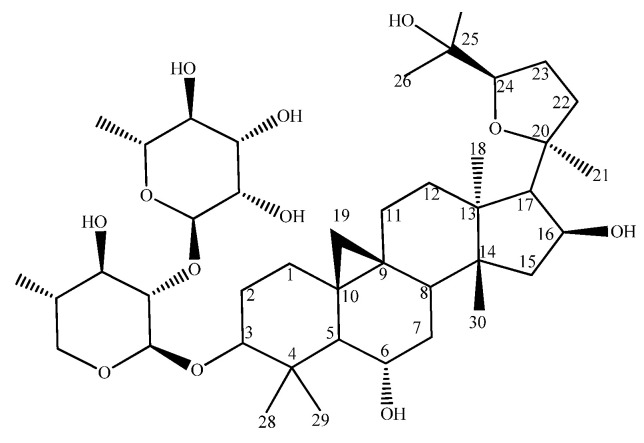
Structure of astrachrysoside A.

Compound **S2** was isolated as a white amorphous powder (20 mg) and had [α]^D^_20_ −2.0698 (c 0.1, MeOH). It was identified via HRESIMS (Figures S30 and S31), 1D, 2D NMR experiments (Figures S11–S27), a GC-MS analysis of the absolute configuration of its sugar moieties (Figure S33) [16], and a comparison to the literature as 3-O-[α-L-rhamnopyranosyl-(1→2)]-β-D-xylopyranosyl]-cycloastragenol (astrachrysoside A) [27] (Figure 2). A major issue of NMR data recorded in methanol-*d*_4_ is that the methine groups C-2 and C-3 of the xylose in compound **S2** have the same values for their carbon and proton resonances, meaning that the point of attachment of the rhamnose cannot be determined via HMBC or NOESY experiments. To circumvent this issue, a second NMR data set of S2 was recorded in pyridine-*d*_5_ and all the resonances were assigned (Table S2). In pyridine, the resonances at C-2 and C-3 in the xylose were no longer identical, and the anomeric proton of the rhamnose gave an unambiguous HMBC correlation to C-2 of the xylose (Figure S18), allowing for a confirmation of the sugar substructure via NMR.

### 3.2. Pharmacological Investigation

Both compounds were tested in vitro for their possible antioxidant and neuroprotective activity in rat brain synaptosomes, mitochondria, and microsomes. 

**Figure 3:**
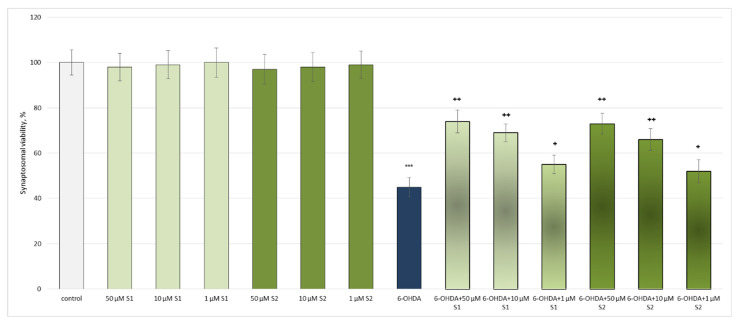
Effects of **S1** and **S2** on the synaptosomal viability, administered alone and in a model of 6-OHDA-induced oxidative stress; *** *p* < 0.001 vs. untreated synaptosomes (control); ^+^ *p* < 0.05; ^++^ *p* < 0.01 vs. 6-OHDA.

**Figure 4:**
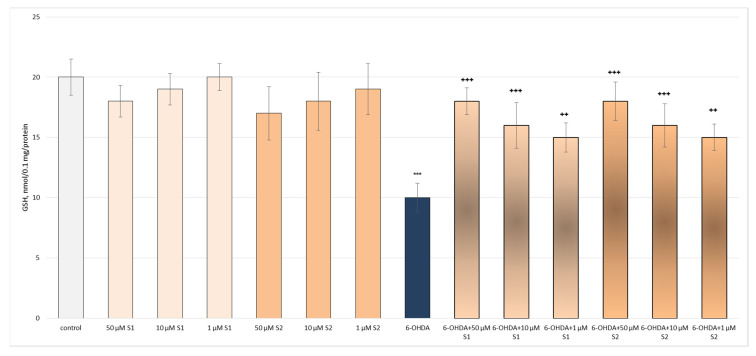
Effects of **S1** and **S2** on GSH level in isolated synaptosomes, administered alone and in a model of 6-OHDA-induced oxidative stress. *** *p* < 0.001 vs. untreated synaptosomes (control); ^++^ *p* < 0.01; ^+++^ *p* < 0.001 vs. 6-OHDA.

6-Hydroxydopamine (6-OHDA, 150 μM, administered alone) showed statistically significant neurotoxic effects. It reduced the synaptosomal viability and GSH levels by 55% and 50%, respectively, compared to the non-treated synaptosomes (control) (Figure 3 and Figure 4).

In the conditions of 6-OHDA-induced oxidative stress, **S1** and **S2** exhibited well-pronounced, concentration-dependent neuroprotective and antioxidant effects, preserving the synaptosomal viability and GSH levels in all the tested concentrations. At the highest tested concentration (50 µM), both **S1** and **S2** had the strongest effects on this model (Figure 3 and Figure 4).

**Figure 5:**
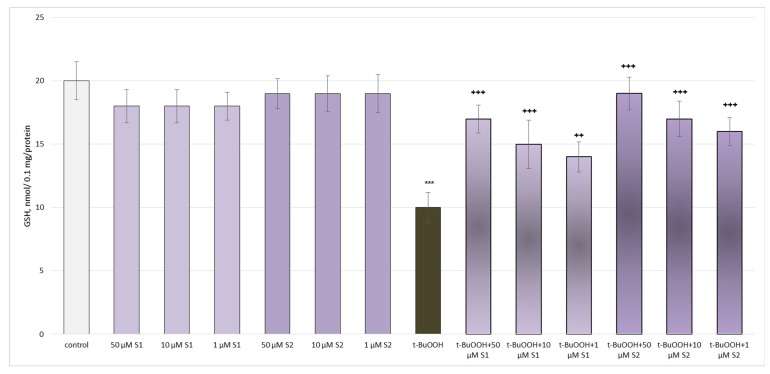
Effects of **S1** and **S2** on GSH level in isolated brain mitochondria, administered alone and in a model of t-BuOOH-induced oxidative stress; *** *p* < 0.001 vs. control (untreated mitochondria); ^++^ *p* < 0.01; ^+++^ *p* < 0.001 vs. t-BuOOH.

**Figure 6:**
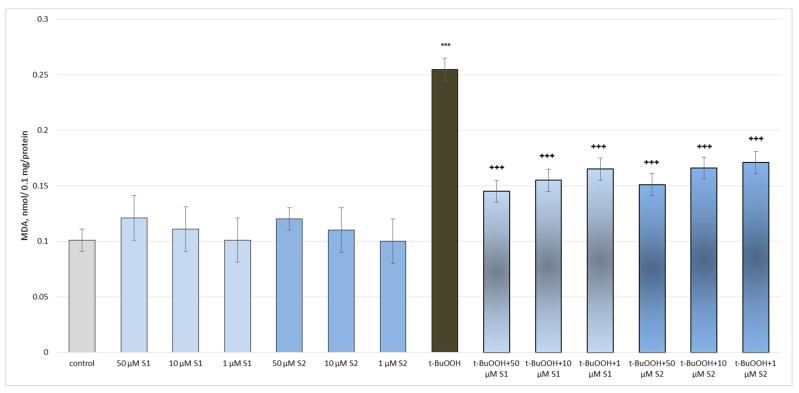
Effects of **S1** and **S2** on MDA production in isolated brain mitochondria, administered alone and in a model of t-BuOOH-induced oxidative stress; *** *p* < 0.001 vs. control (untreated mitochondria); ^+++^ *p* < 0.001 vs. t-BuOOH.

Administered alone, both saponins did not exhibit a statistically significant neurotoxic effect on the mitochondria. Tert-butyl hydroperoxide (t-BuOOH), applied alone, reduced the GSH levels by 50% and increased the MDA production by 152% compared to the untreated mitochondria (control), thus exhibiting a neurotoxic effect (Figure 5 and Figure 6).

Both saponins exhibited a pronounced, concentration-dependent neuroprotective and antioxidant effect on the isolated brain mitochondria in the tert-butyl hydroperoxide-induced oxidative stress model (Figure 5 and Figure 6). The highest tested concentration (50 µM) was again the most effective. Also noteworthy was that the effect in this model on the level of GSH was more pronounced for **S2** than **S1**. For the MDA levels, this was the opposite.

**Figure 7:**
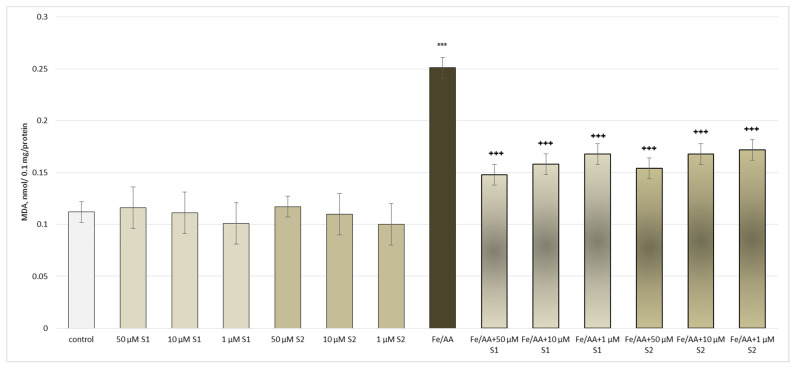
Effects of **S1** and **S2** on MDA production in isolated brain microsomes, administered alone and in a model of non-enzyme-induced lipid peroxidation; *** *p* < 0.001 vs. control (untreated microsomes); ^+++^ *p* < 0.01 vs. Fe/AA.

Administered alone, the saponins did not exhibit a statistically significant pro-oxidant effect on the isolated rat brain microsomes. In the conditions of non-enzymatic lipid peroxidation and at the same concentrations (1 μM, 10 μM, and 50 μM), a significant decrease in the MDA production (by 124%) was observed compared to the non-treated microsomes (control) (Figure 7).

**Figure 8:**
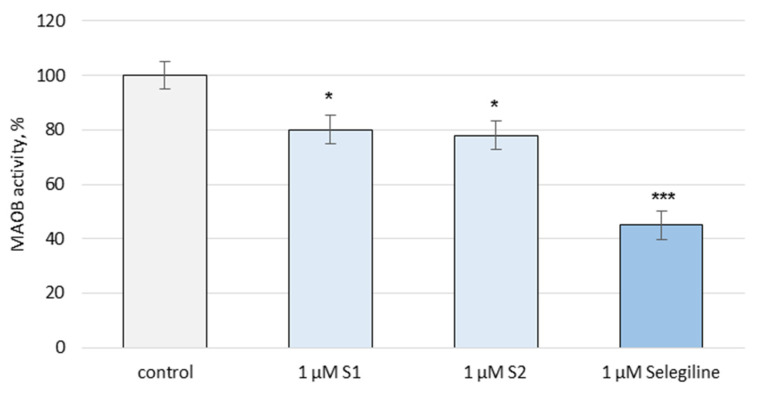
Effects of **S1** and **S2** on the hMAO-B enzyme activity, administered alone; * *p* < 0.05; *** *p* < 0.001 vs. control (untreated hMAO-B).

On the activity of the human recombinant MAO-B enzyme (hMAO-B), the two saponins revealed a weak inhibition. **S1** inhibited the enzyme activity by 20%, and **S2** by 22%, while selegiline, a classical MAO-B inhibitor, decreased the enzyme activity by 55% compared to the control (pure enzyme) (Figure 8).

## 4. Discussion

Cycloartanes are considered to be the predominant type of saponins for Astragalus species, found in the Russian flora. In Bulgaria, these taxa produce mainly oleanane-type saponins [4]. Our studies on the phytochemistry of *A. glycyphyllos* grown in Bulgaria showed that this plant also contains cycloartane saponins. Two of them, isolated from the aerial parts of this species, are new natural compounds—17(R),20(R)-3β,6α,16β-trihydroxycycloartanyl-23-carboxilic acid 16-lactone 3-O-β-D-glucopyranoside [8] and the present 3-O-[α-rhamnopyranosyl-(1→2)]-β-xylopyranosyl]-24-O-α-arabinopyranosyl-3β,6α,16β,24(R),25-pentahydroxy-20R-cycloartane (**S1**), alongside the rare astrachrysoside A (S2). Saponin **S2** had previously been identified in *A. wiedemmanianus* [26], *A. trigonus* [27], and *A. chrysopterus* [28]. Nevertheless, this is the first instance of its isolation from a representative of the Bulgarian flora. Considering that the genus is represented by more than 3500 species worldwide [29], this saponin is rarely occurring. The significant structural difference between the novel saponin **S1** and astrachrysoside A is the sapogenin—cycloasgenin C of **S1** and cycloastragenol of **S2**. Compound **S1** has an aliphphatic side chain attached to C-20, unlike the substituent at C-20 of astrachrysoside A, which is a furan ring. In addition, the available –OH group at C-24 is glycosylated (L-Ara) in compound **S1**. The saponin **S1** has a structural similarity with the triperpenoids previously isolated from the roots of the species, i.e., askendosides C and F—the aglycone is the same and, like askendoside F, is a bisdesmoside [7]. The differences are the type of sugars and the attachment position. 

In vitro systems have an important role in experimental toxicology for studying the biotransformation of substances and establishing the mechanisms by which oxidative stress develops. In addition, they are widely used to study the protective properties of natural compounds. The 6-hydroxydopamine model in isolated brain synaptosomes is a suitable in vitro subcellular system for studying the processes underlying the pathogenesis of degenerative diseases of the nervous system (including Parkinson’s disease). The mechanism by which 6-OHDA neurotoxicity develops is a direct result of its metabolism in neuronal mitochondria. It consists of the production of reactive oxygen species, among other free radicals, and the destruction of nerve endings is thought to be primarily due to the oxidation of 6-OHDA to p-quinone, or free radical or superoxide anion production. It is these reactive intermediates that covalently interact with the nerve terminal, permanently inactivating it [30]. In the synaptosomes, prepared from rat brains with Percoll gradient, both saponins had statistically significant neuroprotective and antioxidant effects in a model of 6-OHDA-induced oxidative stress. This could be connected with their free-radical-scavenging potential, resulting in an elimination of the superoxide anion produced by the p-quinone [30].

There are other reports on the neuroprotective effects of triterpene saponins. Platycodin A, isolated from Platycodi radix, showed neuroprotective activities, increasing the cell viability by about 50% in a model of glutamate-induced toxicity [31]. Several tetracyclic triterpenoid saponins (at a concentration of 10 μM), isolated from Ginseng roots, showed neuroprotective effects on human neuroblastoma SH-SY5Y cells with H_2_O_2_-induced oxidative stress [32]. In vitro and in vivo studies have indicated that NgR1/RhoA/ROCK2 pathway expression regulation is the leading mechanism of action of these saponins [33]. The results obtained correlate with what is known in the literature about the protective effects of other saponins obtained from the species from the genus Astragalus. Other cycloartanes, such as cycloastragenol and astragaloside IV, have been proven as neuroprotectors [34,35]. It was found that astragaloside IV (100 µM) revealed neuroprotective effects in neuronal cell cultures treated with 6-OHDA [36], the same in vitro model of Parkinson’s disease as that used in the present study. In a previous report, a cycloartane saponin, isolated from the aerial parts of *A. glycyphyllos*, displayed a strong neuroprotective effect on the same model at 100 µM [8]. A similar effect was proven for yet another oleanane-type saponin (100 µM) from *A. glycyphylloides* [37].

A suitable model of neuronal oxidative stress is t-BuOOH, which has a mitochondrial and microsomal metabolism, leading to free radicals formed through several steps. In microsomes, when NADPH is absent, the single-electron oxidation of peroxyl radicals occurs. Single-electron reduction to alkoxyl radicals happens in the presence of NADPH. In cells and isolated mitochondria, t-BuOOH forms, via β-cleavage, a methyl radical. All these radicals unlock the process of lipid peroxidation and reduce the level of reduced glutathione [38,39]. In the conditions of oxidative stress induced by t-BuOOH, both saponins tested exhibited statistically significant, concentration-dependent neuroprotective and antioxidant effects. These effects are most likely associated with the metabolism of t-BuOOH at the microsomal and mitochondrial levels, by preserving the reduced glutathione (cell protector) and possible free radical scavenging [38].

The human recombinant MAO-B enzyme is a convenient way of detecting possible inhibitors. The results observed for the isolated saponins were correlated with the data of a previous study on the inhibitory effect of an oleanane saponin from *A. glycyphylloides* [37] and a cycloartane saponin from *A. glycyphyllos* [8], showing again weak inhibitory effects on the hMAO-B enzyme activity.

These findings suggest that the protective effects on synaptosomes, brain mitochondria, and microsomes are not directly linked to direct hMAO-B inhibition. More likely, they are an indirect result of the weak radical-scavenging effects of the saponins.

## 5. Conclusions

From the aerial parts of *A. glycyphyllos,* two cycloartane-type triterpenpoid saponins, 3-O-[α-L-rhamnopyranosyl-(1→2)]-β-D-xylopyranosyl]-24-O-α-L-arabinopyranosyl-3β,6α,16β,24(R),25-pentahydroxycycloartane and astrachrysoside A, were isolated via various chromatographic techniques. They were structurally elucidated using extensive HRESIMS, NMR, and GC-MS experiments. Both compounds displayed significant in vitro neuroprotective and antioxidant effects in a 6-OHDA-induced neurotoxicity model on isolated brain synaptosomes, t-BuOOH-induced oxidative stress in brain mitochondria, and on isolated rat brain microsomes, in a model of lipid peroxidation (non-enzyme-induced). The observed effects were concentration-dependant. Both saponins revealed a weak inhibitory effect on the activity of hMAO-B in comparison with selegiline. These results suggest that *A. glycyphyllos* saponins could be considered as perspective for future research.

## Figures and Tables

**Table 1:** ^1^H NMR spectroscopic data (700 MHz, *J* in Hz) and ^13^C NMR spectroscopic data (175 MHz) of **S1** in methanol-*d*_4._

Position	δ_C_ [ppm], Type	*δ*_H_ [ppm], (*J* in Hz)
1	33.5, CH_2_	1.54, t (13.0) 1.21
2	30.7, CH_2_	1.91, m 1.66
3	89.3, CH	3.18, d (~13)
4	43.2, C	-
5	54.8, CH	1.35, d (9.9)
6	69.6, CH	3.44, m
7	38.8, CH_2_	1.33 1.45
8	48.8, CH	1.79, dd (12.0, 4.2)
9	22.1, C	-
10	30.4, C	-
11	27.0, CH_2_	1.191.97
12	33.9, CH_2_	1.66 1.60, td (12.0, 3.2)
13	46.4, C	-
14	47.4, C	-
15	48.8, CH_2_	1.99 1.38
16	73.1, CH	4.40, m
17	58.1, CH	1.66
18	19.2, CH_3_	1.13 s
19	31.9, CH_2_	0.37, d (4.0) 0.52, d (4.0)
20	32.5, CH	1.75
21	18.7, CH_3_	0.93, d (6.5)
22	34.6, CH_2_	2.08, brt (12.5) 0.96
23	30.4, CH_2_	1.701.25
24	92.5, CH	3.30
25	74.9, C	-
26	26.4, CH_3_	1.15, s
27	24.0, CH_3_	1.18, s
28	16.8, CH_3_	1.02, s
29	28.6, CH_3_	1.28, s
30	20.4, CH_3_	1.54, t (13.0) 1.21
Xyl-I		
1	106.2, CH	4.37, d (~6.9)
2	78.9, CH	3.42
3	78.8, CH	3.42
4	71.6, CH	3.47
5	66.5, CH_2_	3.17, t (10.6) 3.84, dd (11.0, 5.3)
Rha-II		
1	102.1, CH	5.33, brs
2	72.2, CH	3.94, brs
3	72.2, CH	3.74, dd (9.3, 2.8)
4	74.0, CH	3.38, t (9.6)
5	70.1, CH	3.98, dq (9.4, 6.1)
6	18.1, CH_3_	1.23, d (6.3)
Ara-III		
1	107.4, CH	4.38, d (7.5)
2	73.7, CH	3.58
3	75.0, CH	3.49
4	70.2, CH	3.78, brs
5	68.0, CH_2_	3.56 3.87, dd (12.5, 1.8)

**Table 2:** Comparison of ^13^C chemical shifts of S1 with data of glycosylated sidechains which have different configuration at C-24. All data were recorded in methanol-*d*_4_.

Atom	S1	24 S *A. brachypterus* [25]	24 R *A. stereocalyx* [26]
	δ_C_ [ppm]	δ_C_ [ppm]	δ_C_ [ppm]
C-21	18.7	17.5	18.2
C-20	32.5	30.9	32.4
C-22	34.6	33.0	34.2
C-23	30.4	29.4	30.1
C-24	92.5	89.7	91.8
C-25	74.9	73.5	75.0

## Data Availability

Data connected with this study are freely available from the corresponding author, upon reasonable written request. The data are not publicly available due to the large volume of the files.

## Supplementary Materials

The following supporting information can be downloaded at: https://www.mdpi.com/article/10.3390/metabo13070857/s1, Table S1. ^1^H NMR spectroscopic data (700 MHz, *J* in Hz) and ^13^C NMR spectroscopic data (175 MHz) of **S1** in methanol-*d*_4_; Table S2. ^1^H NMR spectroscopic data (700 MHz, *J* in Hz) and ^13^C NMR spectroscopic data (175 MHz) of compound **S2** in methanol-*d*_4_ and pyridine-*d*_5_.; Figure S1: ^1^H-NMR spectrum (700 MHz, methanol-*d*_4_) of compound **S1**; Figure S2: Expansion of the ^1^H-NMR spectrum of compound **S1** (sugar region); Figure S3: Expansion of the ^1^H-NMR spectrum of compound **S1** (aglycone region); Figure S4: ^13^C-NMR spectrum (175 MHz, methanol-*d*_4_) of compound **S1**; Figure S5: COSY spectrum (700 MHz, methanol-*d*_4_) of compound **S1**; Figure S6: HSQC spectrum (700/175 MHz, methanol-*d*_4_) of compound **S1**; Figure S7: Expansion of the sugar region of the HSQC spectrum of compound **S1**; Figure S8: HMBC spectrum (700/175 MHz, methanol-*d*_4_) of compound **S1**; Figure S9: Expansion of the sugar region of the HMBC spectrum of compound **S1**; Figure S10: ROESY-spectrum (700 MHz, methanol-*d*_4_) of compound **S1**; Figure S11: ^1^H-NMR spectrum (700 MHz, pyridine-*d*_5_) of compound **S2**; Figure S12: Expansion of the ^1^H-NMR (700 MHz, pyridine-*d*_5_) spectrum of compound **S2**; Figure S13: Expansion of the ^1^H-NMR (700 MHz, pyridine-*d*_5_) spectrum of compound **S2**; Figure S14: ^13^C-NMR spectrum (175 MHz, pyridine-*d*_5_) of compound **S2**; Figure S15: COSY spectrum (700 MHz, pyridine-*d*_5_) of compound **S2**; Figure S16: HSQC spectrum (700/175 MHz, pyridine-*d*_5_) of compound **S2**; Figure S17: HMBC spectrum (700/175 MHz, pyridine-*d*_5_) of compound **S2**; Figure S18: Expansion of the sugar region of the HMBC spectrum (700/175 MHz, pyridine-*d*_5_) of compound **S2**; Figure S19: ROESY spectrum (700 MHz, pyridine-*d*_5_) of compound **S2**; Figure S20: ^1^H-NMR spectrum (700 MHz, methanol-*d*_4_) of compound **S2**; Figure S21: Expansion of the ^1^H-NMR (700 MHz, methanol-*d*_4_) spectrum of compound **S2**; Figure S22: Expansion of the ^1^H-NMR (700 MHz, methanol-*d*_4_) spectrum of compound **S2**; Figure S23: ^13^C-NMR spectrum (175 MHz, methanol-*d*_4_) of compound **S2**; Figure S24: COSY spectrum (700 MHz, methanol-*d*_4_) of compound **S2**; Figure S25: HSQC spectrum (700/175 MHz, methanol-*d*_4_) of compound **S2**; Figure S26: HMBC spectrum (700/175 MHz, methanol-*d*_4_) of compound **S2**; Figure S27: ROESY spectrum (700 MHz, pyridine-*d*_5_) of compound **S2**; Figure S28: HRESIMS of **S1** in negative mode; Figure S29: HRESIMS of **S1** in positive mode; Figure S30: HRESIMS of **S2** in negative mode; Figure S31: HRESIMS of **S2** in positive mode. Figure S32: GC chromatogram of the monosaccharides of S1. Figure S33: GC chromatogram of the monosaccharides of S2.
