# Supplementary material for: Cycloartane Saponins from Astragalus glycyphyllos and Their In Vitro Neuroprotective, Antioxidant, and hMAO-B-Inhibiting Effects
Source: Metabolites. 2023 Jul 19;13(7):857. doi: 10.3390/metabo13070857 (PMC10385106; doi:10.3390/metabo13070857)
Supplement: Supplementary file 1 [file metabolites-13-00857-s001.zip › metabolites-2492141-supplementary.pdf]

Cycloartane Saponins from Astragalus glycyphyllos and Their In Vitro Neuroprotective, Antioxidant, and hMAO-B-Inhibiting Effects

Ivan Stambolov [1], Aleksandar Shkondrov [1], Olaf Kunert [2], Franz Bucar [3], Magdalena Kondeva-Burdina [4] and Ilina Krasteva [1,*]

1 Department of Pharmacognosy, Faculty of Pharmacy, Medical University of Sofia, 2 Dunav st., 1000 Sofia, Bulgaria; istambolov@pharmfac.mu-sofia.bg (I.S.); shkondrov@pharmfac.mu-sofia.bg (A.S.)
2 Department of Pharmaceutical Chemistry, Institute of Pharmaceutical Sciences, University of Graz, Universitätsplatz 1, A-8010 Graz, Austria; olaf.kunert@uni-graz.at
3 Department of Pharmacognosy, Institute of Pharmaceutical Sciences, University of Graz, Beethovenstrasse 8, A-8010 Graz, Austria; franz.bucar@uni-graz.at
4 Laboratory of Drug Metabolism and Drug Toxicity, Department of Pharmacology, Pharmacotherapy and Toxicology, Faculty of Pharmacy, Medical University of Sofia, 2 Dunav st., 1000 Sofia, Bulgaria; mkondeva@pharmfac.mu-sofia.bg
* Correspondence: ikrasteva@pharmfac.mu-sofia.bg; Tel.: +359-2-9236-552

Supplementary material

Table S1. ^{1}H NMR spectroscopic data (700 MHz, *J* in Hz) and ^{13}C NMR spectroscopic data (175 MHz) of **S1** in methanol-d_4

**position**	**δ_C, type**	**δ_H**	HMBC correlations δ_C, (atom)	ROESY cross peaks δ_H, (atom)
1	33.5, CH_2	1.54, t (13.0)<br>1.21	31.9 (C-19), 30.7 (C-2)	
2	30.7, CH_2	1.91, m<br>1.66		
3	89.3, CH	3.18, d (~13)	106.2 (Xyl-1), 43.3 (C-4), 28.6 (C-29), 16.8 (C-28)	1.91 (H-2), 1.54 (H-1), 1.35 (H-5), 1.28 (H-29)
4	43.2, C	-		

5	54.8, CH	1.35, d (9.9)		
6	69.6, CH	3.44, m	54.8 (C-5), 48.8 (C-8), 43.2 (C-4)	1.79 (H-8), 1.45 (H-7), 0.53 (H-19)
7	38.8, CH_2	1.45<br>1.33		
8	48.8, CH	1.79, dd (12.0, 4.2)	69.6 (C-6), 48.8 (C-15), 47.7 (C-14), 38.8 (C-7), 31.9 (C-19), 22.1 (C-9), 20.5 (C-30)	3.44 (H-6), 1.45 (H-7), 0.53 (H-19)
9	22.1, C	-		
10	30.1, C	-		
11	27.0, CH_2	1.97<br>1.19		
12	33.9, CH_2	1.66<br>1.60, td (12.0, 3.2)	27.0 (C-11)	
13	46.4, C	-		
14	47.4, C	-		
15	48.8, CH_2	1.99<br>1.38	73.1 (C-16), 58.1 (C-17), 47.4 (C-14), 46.4 (C-13), 20.4 (C-30)	

16	73.1, CH	4.40, m	46.4 (C-13)	2.09 (H-22), 1.99 (H-15), 1.66 (H-17), 0.94 (H-30)
17	58.1, CH	1.66		
18	19.2, CH_3	1.13 s	58.1 (C-17), 47.4 (C-14), 46.4 (C-13), 33.9 (C-12)	1.79 (H-8), 0.53 (H-19), 0.37 (H-19)
19	31.9, CH_2	0.52, d (4.0)<br>0.37, d (4.0)	54.8 (C-5), 48.8 (C-8), 33.5 (C-1), 30.4 (C-10), 27.0 (C-11), 22.1 (C-9)	3.44 (H-6), 1.79 (H-8), 1.21 (H1), 1.02 (H-28)
20	32.5, CH	1.75	58.1 (C-17), 34.6 (C-22), 32.5 (C-20)	
21	18.7, CH_3	0.93, d (6.5)		1.66 (H-17)
22	34.6, CH_2	2.08, brt (12.5)<br>0.96	92.5 (C-24), 58.1 (C-17), 32.5 (C-20), 30.4 (C-23), 18.7 (C-21)	
23	30.4, CH_2	1.70<br>1.25		
24	92.5, CH	3.30	107.3 (Ara-1), 74.9 (C-25), 34.6 (C-22), 30.4 (C-23), 26.4 (C-26), 24.0 (C-27)	4.38 (Ara-1), 1.70 (H-23)
25	74.9, C	-		

26	26.4, CH_3	1.15, s	92.5 (C-24), 74.9 (C-25), 24.0 (C-27)	
27	24.0, CH_3	1.18, s	92.5 (C-24), 74.9 (C-25), 26.4 (C-26)	
28	16.8, CH_3	1.02, s	89.3 (C-3), 54.8 (C-5), 43.2 (C-4), 28.6 (C-29)	
29	28.6, CH_3	1.28, s	89.3 (C-3), 54.8 (C-5), 43.2 (C-4), 16.8 (C-28)	
30	20.4, CH_3	0.94, s	58.1 (C-17), 34.6 (C-22), 32.5 (C-20)	1.99 (H-15), 1.33 (H-7)
Xyl				
1	106.2, CH	4.37, d (~6.9)	89.3 (C-3), 78.8 (Xyl-3), 66.5 (Xyl-5)	
2	78.9, CH	3.42		
3	78.8, CH	3.42		
4	71.6, CH	3.47		
5	66.5, CH_2	3.84, dd (11.0, 5.3)<br>3.17, t (10.6)	106.2 (Xyl-1), 78.8 (Xyl-3), 71.6 (Xyl-4)	

Rha				
1	102.1, CH	5.33, brs	78.8 (Xyl-2), 72.2 (Rha-2), 70.1 (Rha-5)	3.94 (Rha-2), 3.42 (Xyl-2/3), 1.02 (H-28)
2	72.2, CH	3.94, brs		
3	72.2, CH	3.74, dd (9.3, 2.8)		
4	74.0, CH	3.38, t (9.6)		
5	70.1, CH	3.98, dq (9.4, 6.1)		
	18.1, CH_3	1.23, d (6.3)	74.0 (Rha-4), 70.1 (Rha-5)	
Ara				
1	107.4, CH	4.38, d (7.5)	92.5 (C-24), 73.6 (Ara-2), 68.0 (Ara-5)	
2	73.7, CH	3.58		
3	75.0, CH	3.49		
4	70.2, CH	3.78, brs		
5	68.0, CH_2	3.87, dd (12.5, 1.8) 3.56	107.4 (Ara-1), 75.0 (Ara-3), 70.2 (Ara-4)	

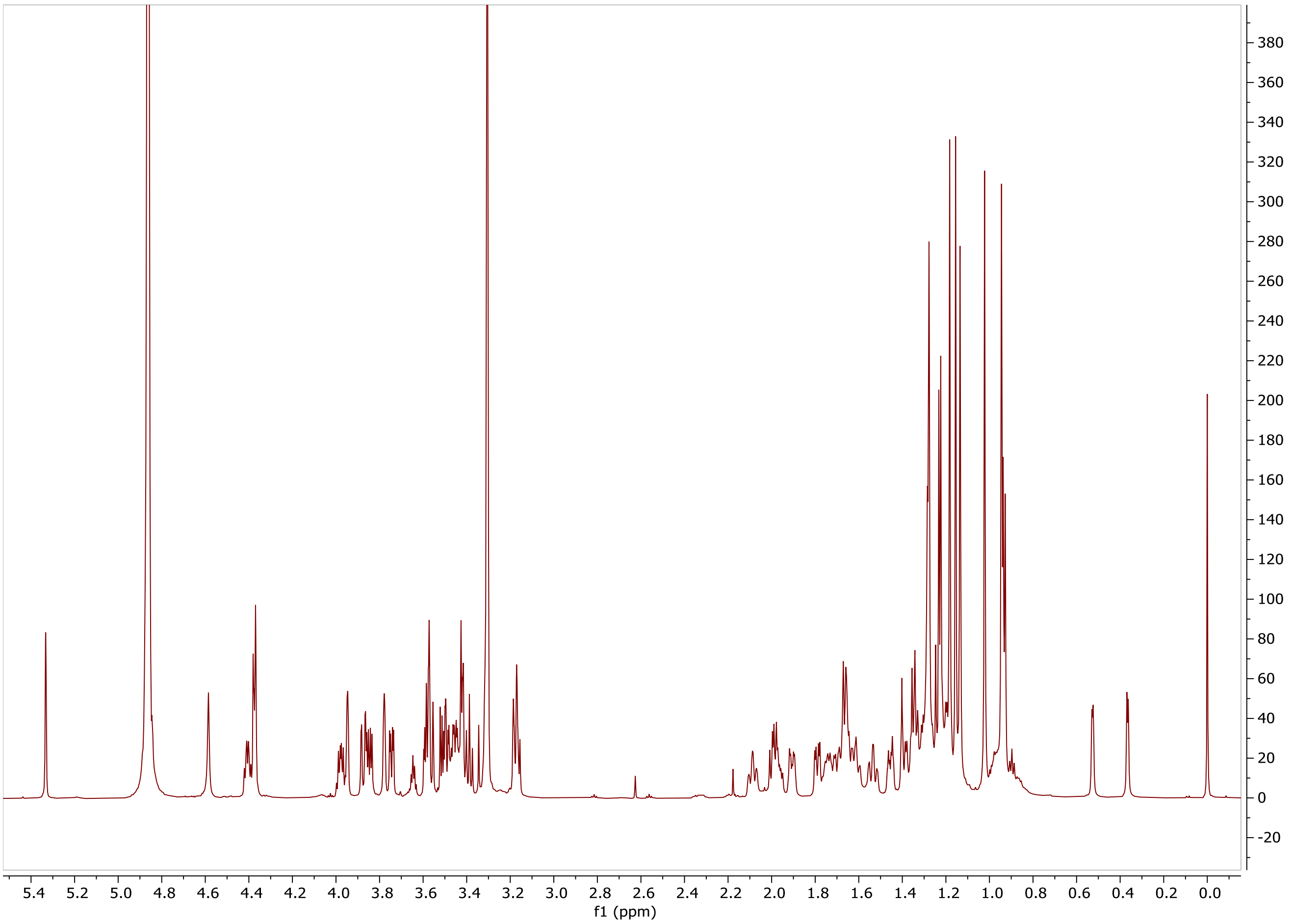

Figure S1: ^{1}H-NMR spectrum (700 MHz, methanol-d_4) of compound **S1**.

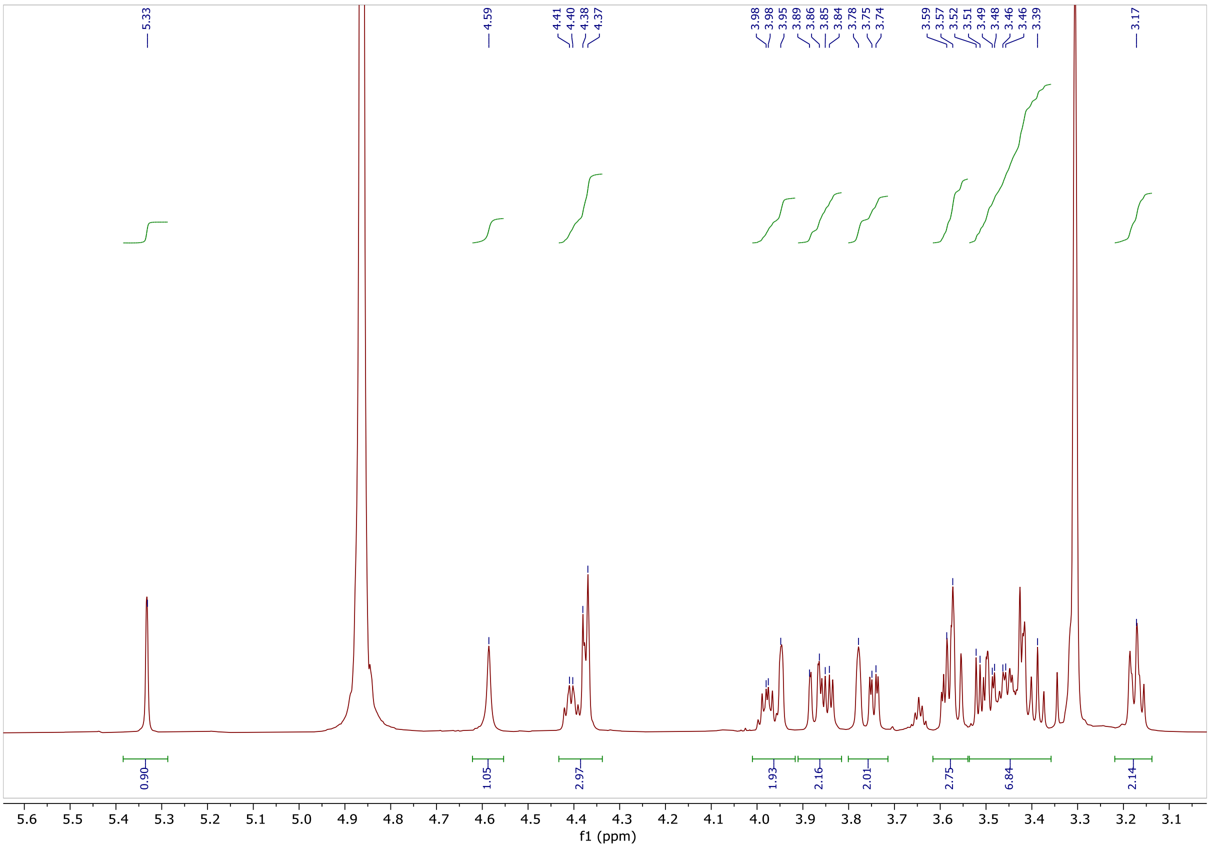

Figure S2: Expansion of the ^{1}H-NMR spectrum of compound **S1** (sugar region).

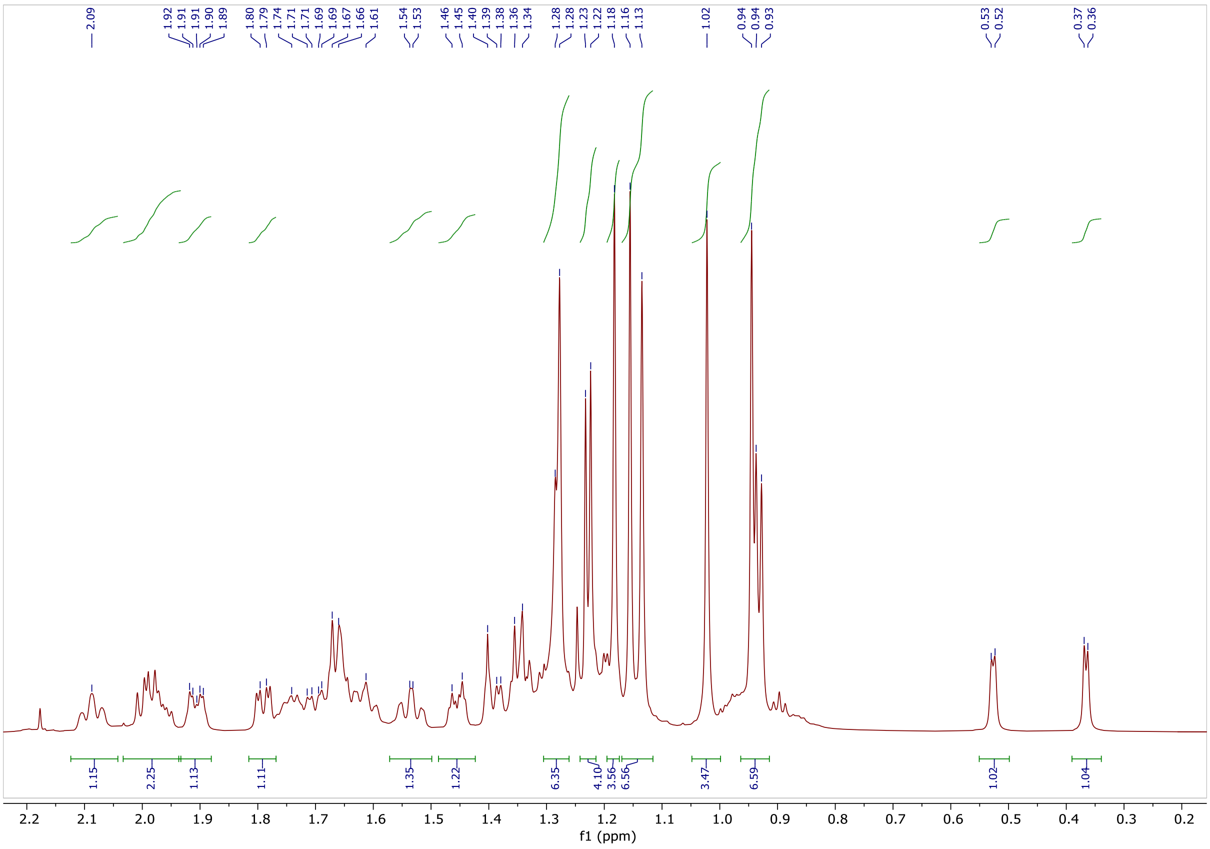

Figure S3: Expansion of the ^{1}H-NMR spectrum of compound **S1** (aglycon region).

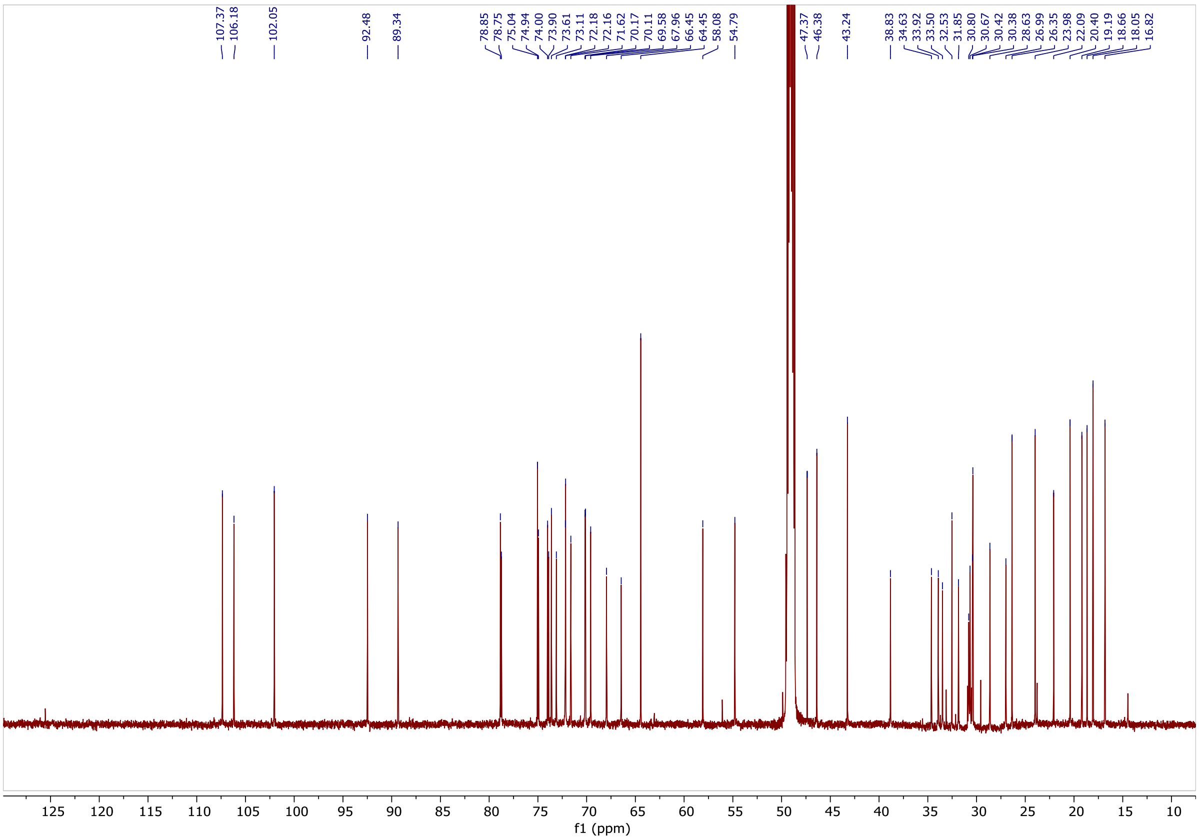

Figure S4: ^{13}C-NMR spectrum (175 MHz, methanol-d_4) of compound **S1**.

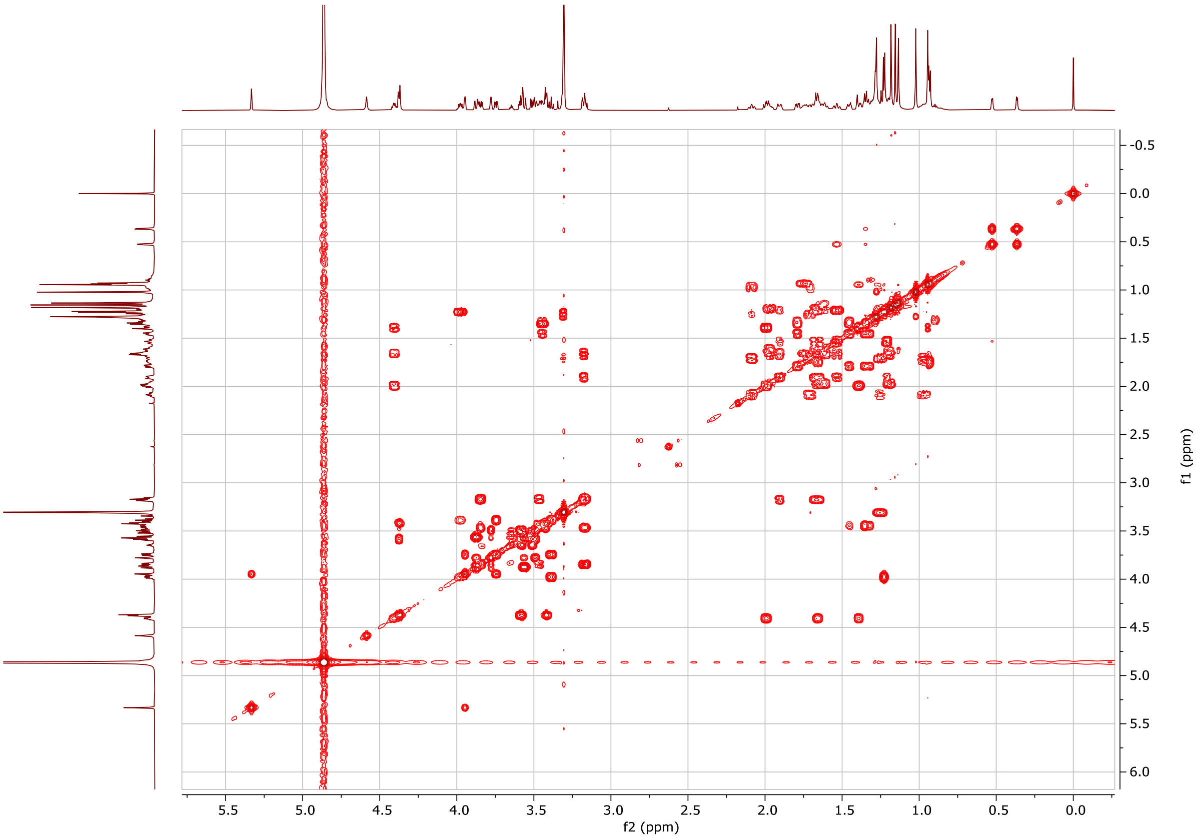

Figure S5: COSY spectrum (700 MHz, methanol-d_4) of compound **S1**.

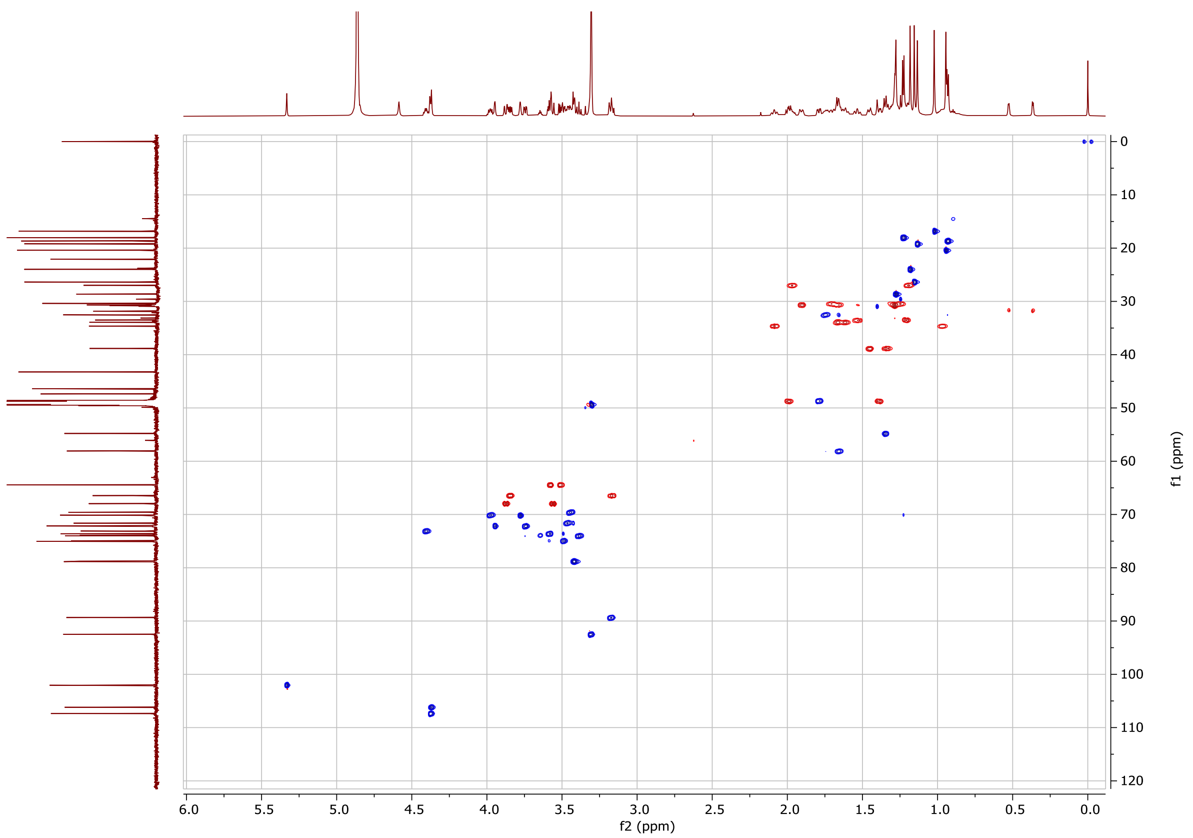

Figure S6: HSQC spectrum (700/175 MHz, methanol-d_4) of compound **S1**.

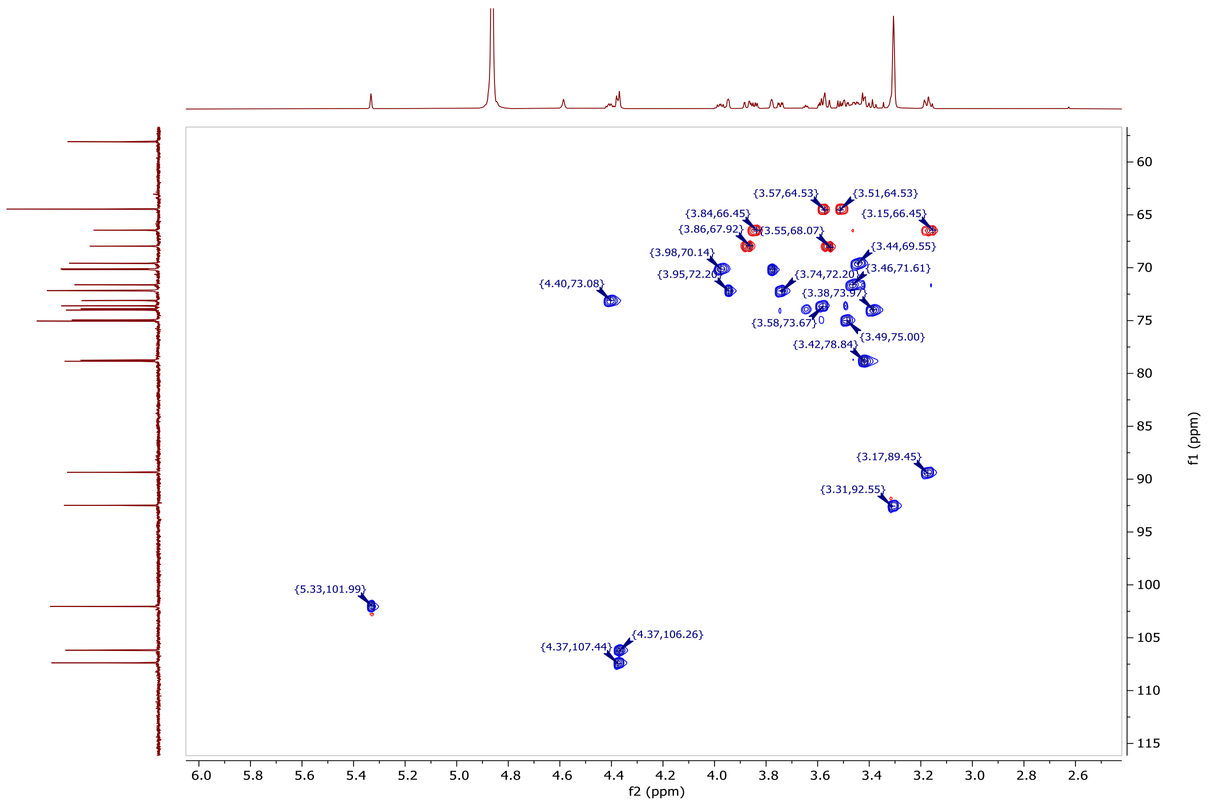

Figure S7: Expansion of the sugar region of the HSQC spectrum of compound **S1**.

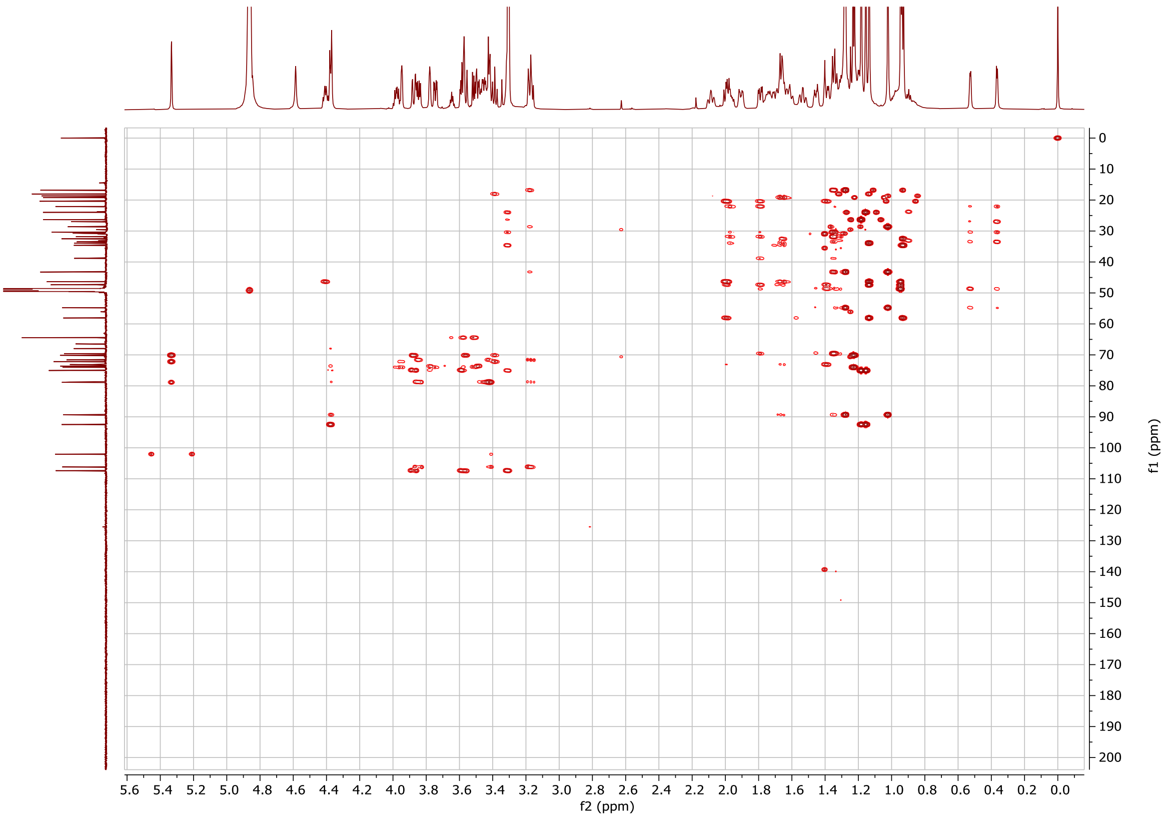

Figure S8: HMBC spectrum (700/175 MHz, methanol-d_4) of compound **S1**.

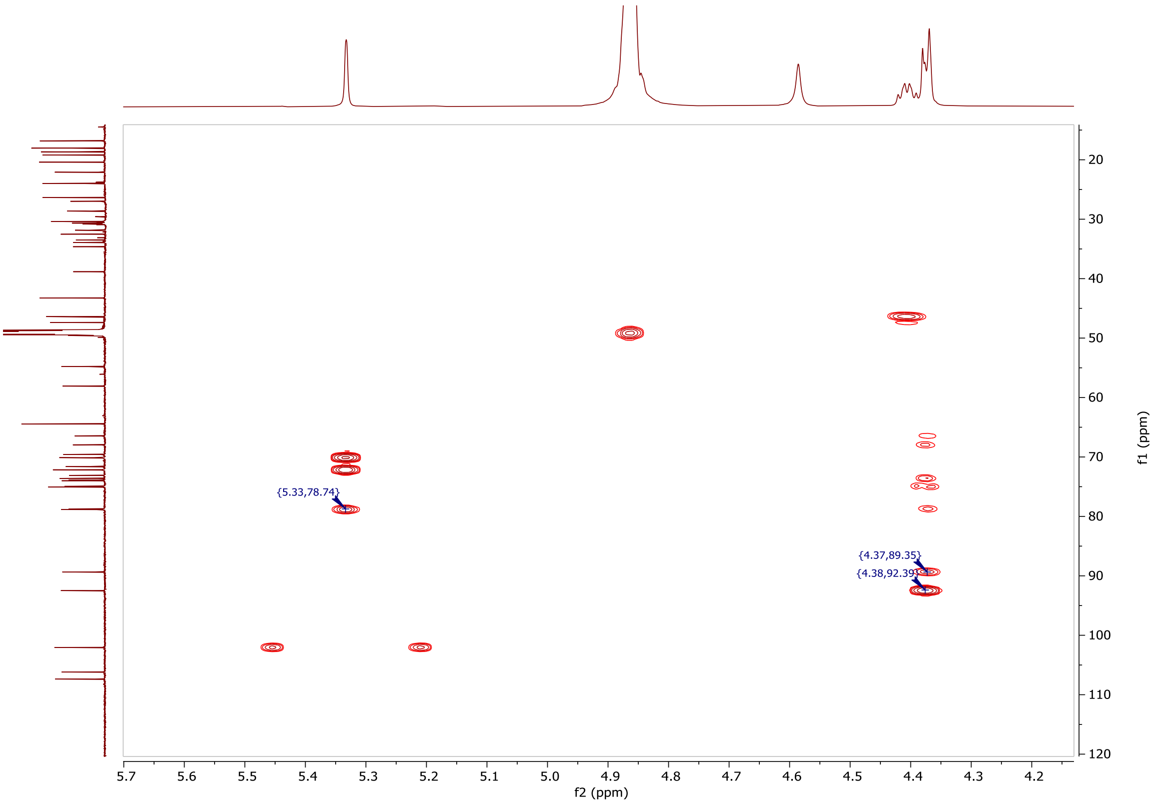

Figure S9: Expansion of the sugar region of the HMBC spectrum of compound **S1**.

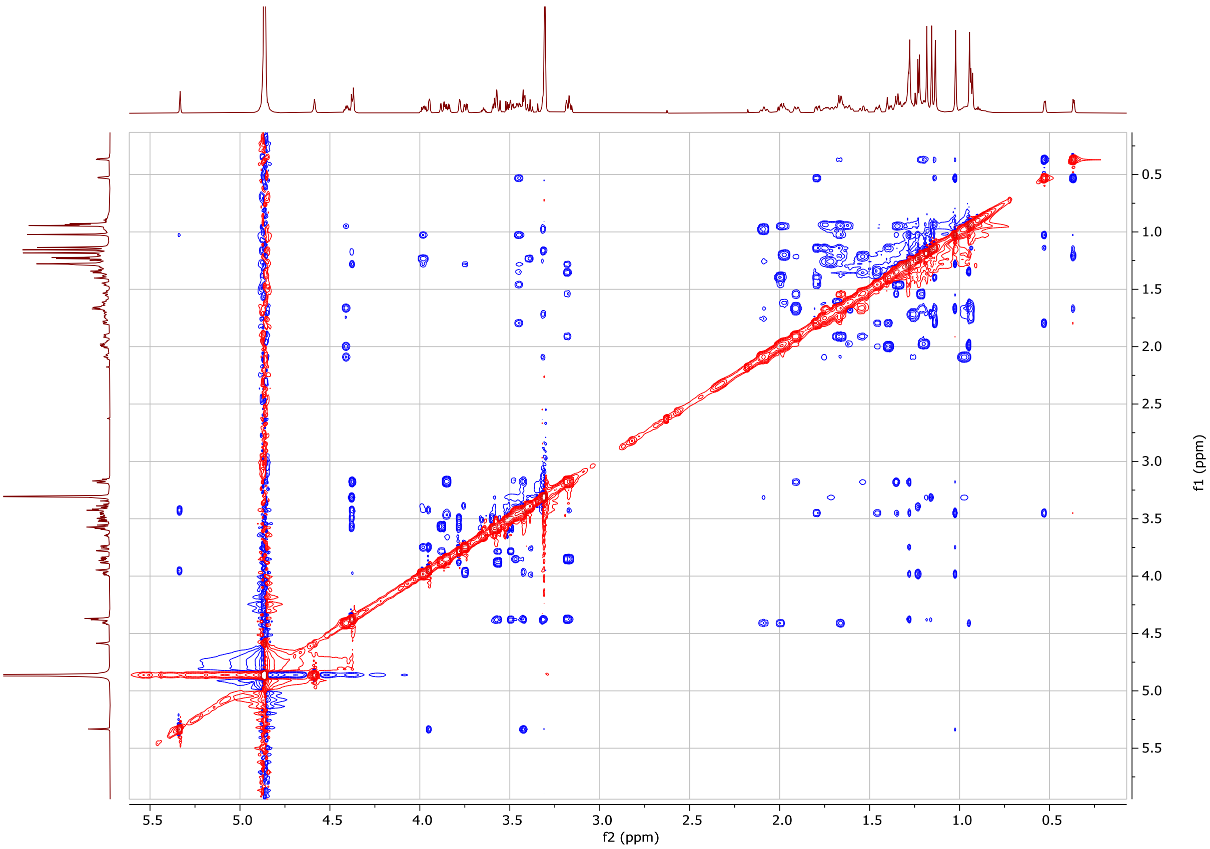

Figure S10: ROESY-spectrum (700 MHz, methanol-*d*$_4$) of compound **S1**.

Table S2. ^{1}H NMR spectroscopic data (700 MHz, *J* in Hz) and ^{13}C NMR spectroscopic data (175 MHz) of compound **S2** in methanol-d_4 and pyridine-d_5.

	**S2 in MeOH-*d*4**			**S2 in pyridine-*d*5**	
**position**	**δ_C, type**	**δ_H**		**δ_C, type**	**δ_H**
1	33.5, CH_2	1.54<br>1.21		32.6, CH_2	1.62<br>1.23
2	30.7, CH_2	1.91<br>1.66		30.7, CH_2	1.91<br>1.66
3	89.3, CH	3.18		89.3, CH	3.58
4	43.2, C	-		42.7, C	-
5	54.8, CH	1.35		54.2, CH	1.73
6	69.6, CH	3.45		67.8, CH	3.77
7	39.9, CH_2	1.35<br>1.46		38.6, CH_2	1.67<br>1.84
8	48.8, CH	1.80		46.9, CH	1.94
9	21.8, C	-		20.8, C	-
10	30.7, C	-		32.6, C	-
11	27.0, CH_2	2.01<br>1.22		26.2, CH_2	1.91<br>1.20
12	33.9, CH_2	1.68<br>1.63		33.4, CH_2	1.64<br>1.59
13	46.0, C	-		46.1, C	-
14	47.1, C	-		45.0, C	-
15	48.8, CH_2	1.95<br>1.40		48.8, CH_2	2.12<br>1.76
16	73.1, CH	4.65		73.4, CH	5.03
17	59.1, CH	2.36		58.4, CH	2.54
18	22.0, CH_3	1.26		21.4, CH_3	1.42

19	32.3, CH_2	0.55<br>0.37		30.4, CH_2	0.58<br>0.28
20	88.4, C	-		87.3, C	-
21	28.5, CH_3	1.21		28.6, CH_3	1.32
22	35.5, CH_2	2.16<br>1.64		34.9, CH_2	1.67<br>3.11
23	26.8, CH_2	2.04<br>2.01		26.4, CH_2	2.35<br>2.05
24	82.7, CH	3.75		81.7, CH	3.89
25	72.5, C	-		71.3, C	-
26	26.7, CH_3	1.12		27.1, CH_3	1.31
27	27.7, CH_3	1.25		28.2, CH_3	1.54
28	28.7, CH_3	1.28		28.9, CH_3	1.98
29	16.8, CH_3	1.02		16.8, CH_3	1.52
30	20.6, CH_3	0.99		20.8, CH_3	1.01
Xyl					
1	106.2, CH	4.37		106.1, CH	4.91
2	78.8*, CH	3.43		78.2, CH	4.30
3	78.9*, CH	3.43		79.5, CH	4.17
4	71.6, CH	3.47		71.5, CH	4.17
5	66.5, CH_2	3.85<br>3.17		66.9, CH_2	4.33<br>3.67
Rha					
1	102.1, CH	5.33		102.1, CH	6.56
2	72.1, CH	3.95		72.5, CH	4.89
3	72.1, CH	3.74		72.5, CH	4.75

4	74.0, CH	3.38		74.2, CH	4.38
5	70.1, CH	3.97		69.8, CH	4.90
	18.1, CH_3	1.23		18.8, CH_3	1.76

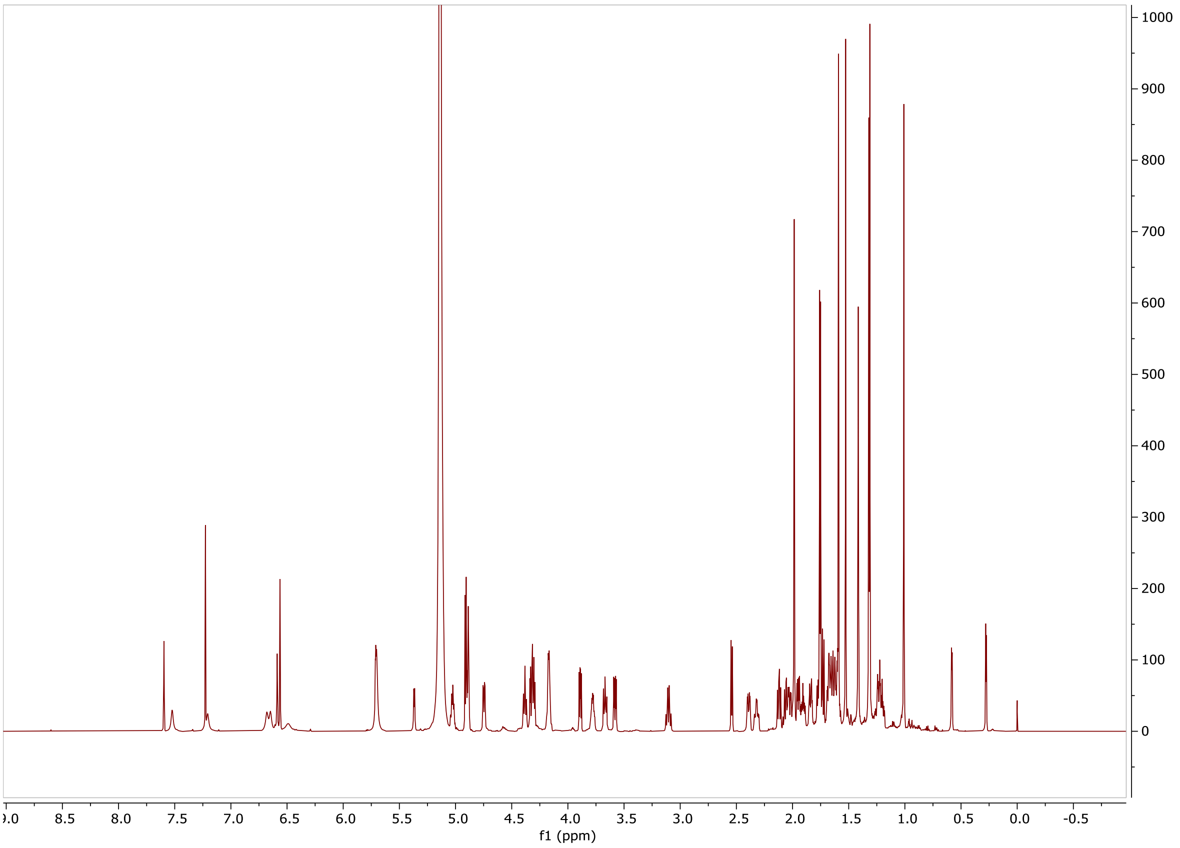

Figure S11: ^{1}H-NMR spectrum (700 MHz, pyridine-d_5) of compound **S2**.

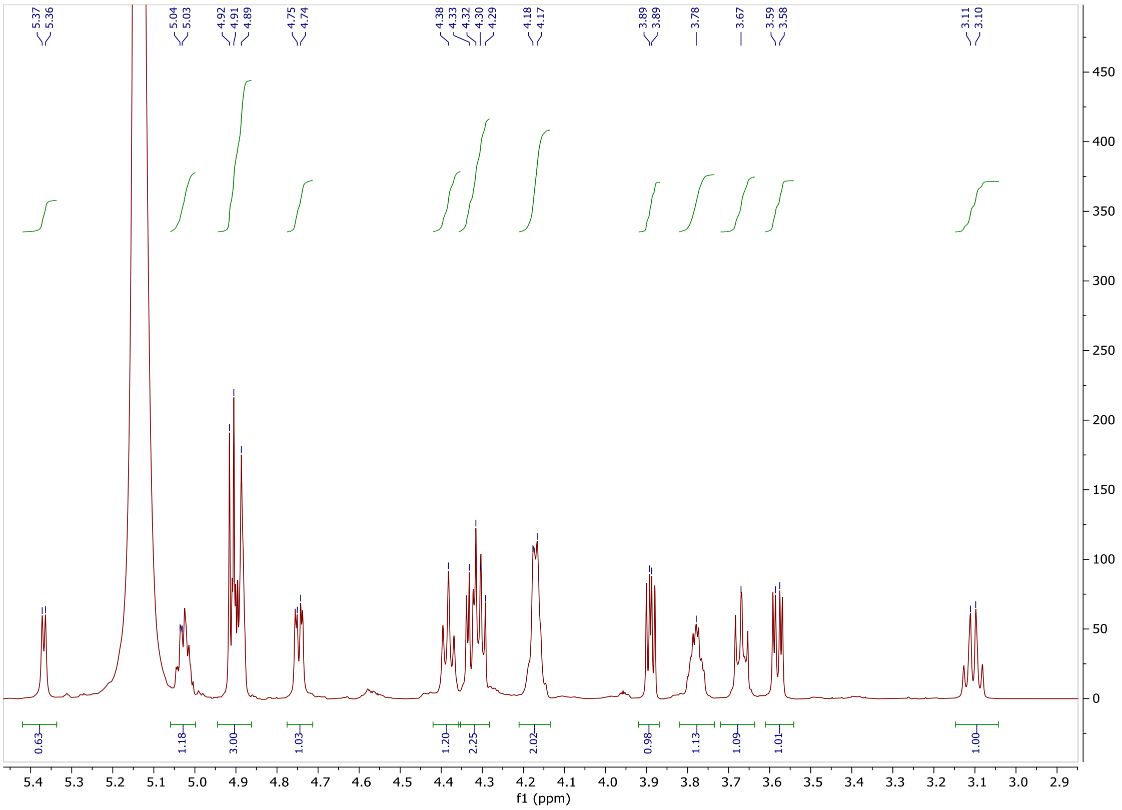

Figure S12: Expansion of the ^{1}H-NMR (700 MHz, pyridine-d_5) spectrum of compound **S2.**

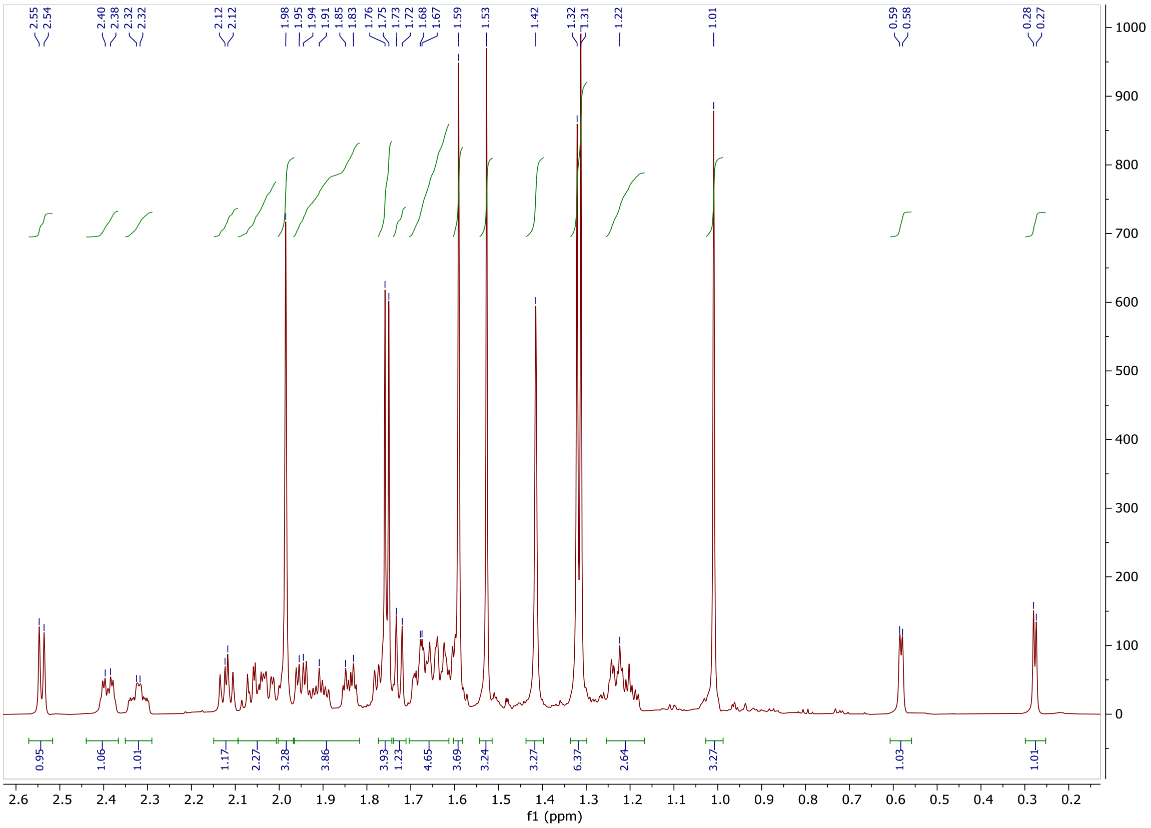

Figure S13: Expansion of the ^{1}H-NMR (700 MHz, pyridine-d_5) spectrum of compound **S2**.

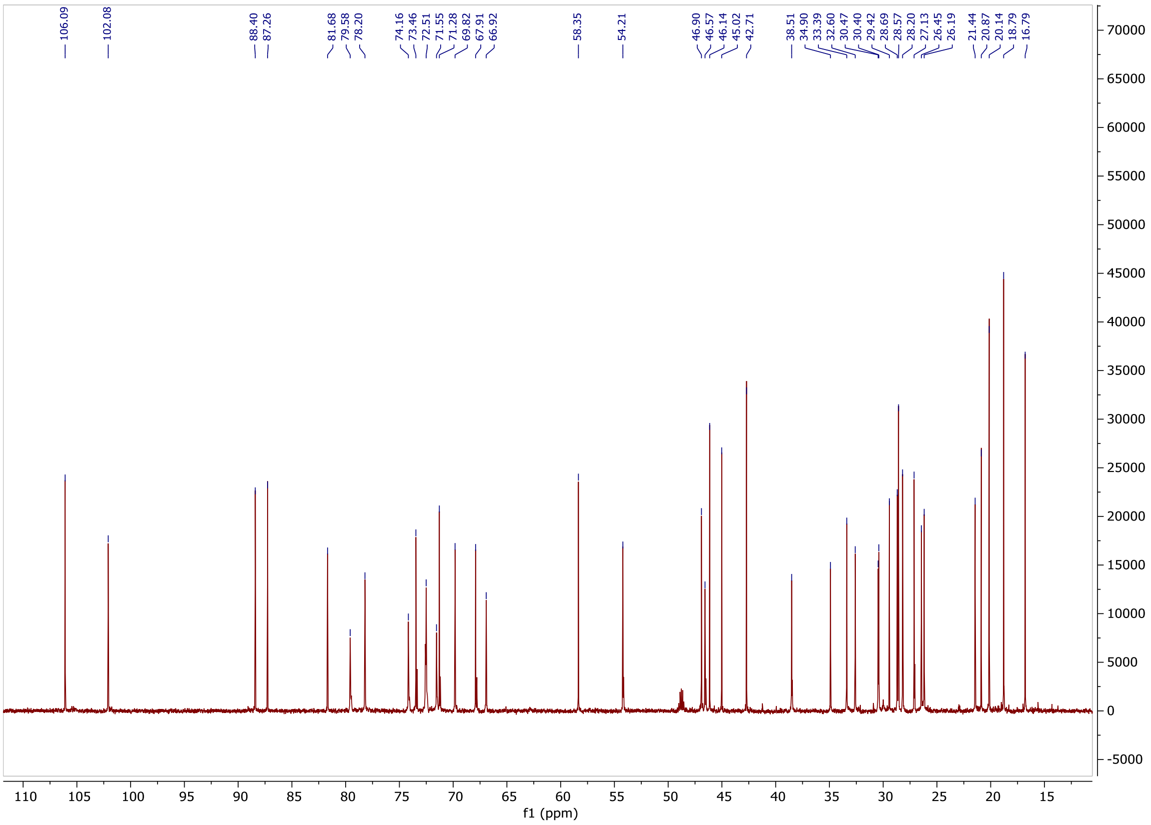

Figure S14: ^{13}C-NMR spectrum (175 MHz, pyridine-d_5) of compound **S2**.

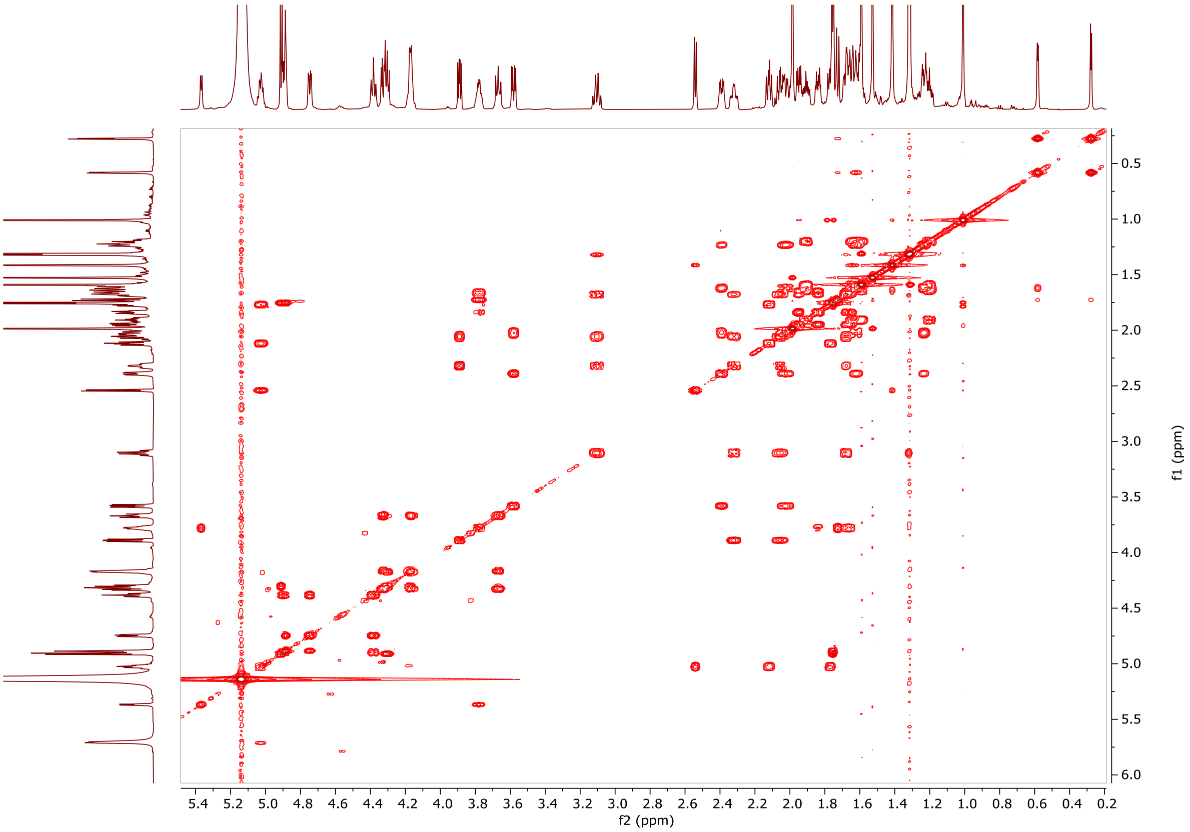

Figure S15: COSY spectrum (700 MHz, pyridine-d_5) of compound **S2**.

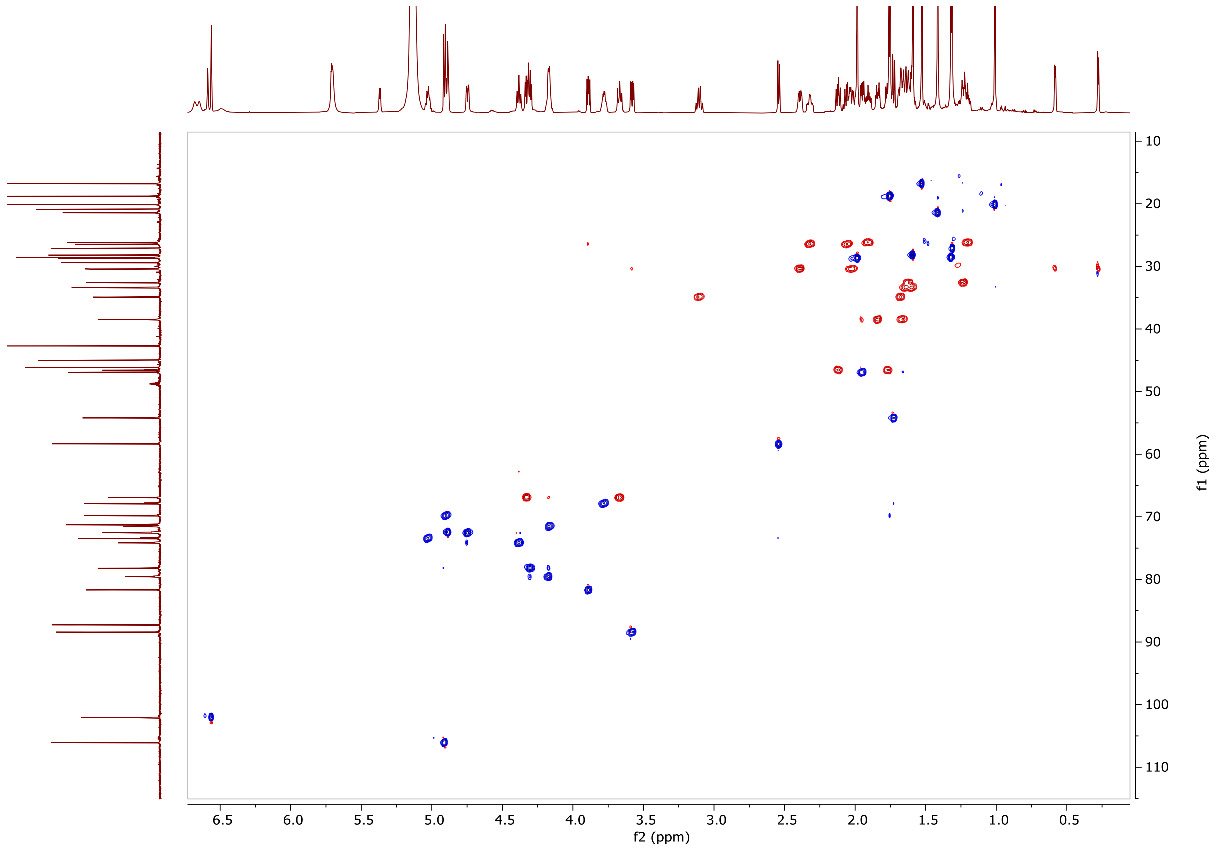

Figure S16: HSQC spectrum (700/175 MHz, pyridine-d_5) of compound **S2**.

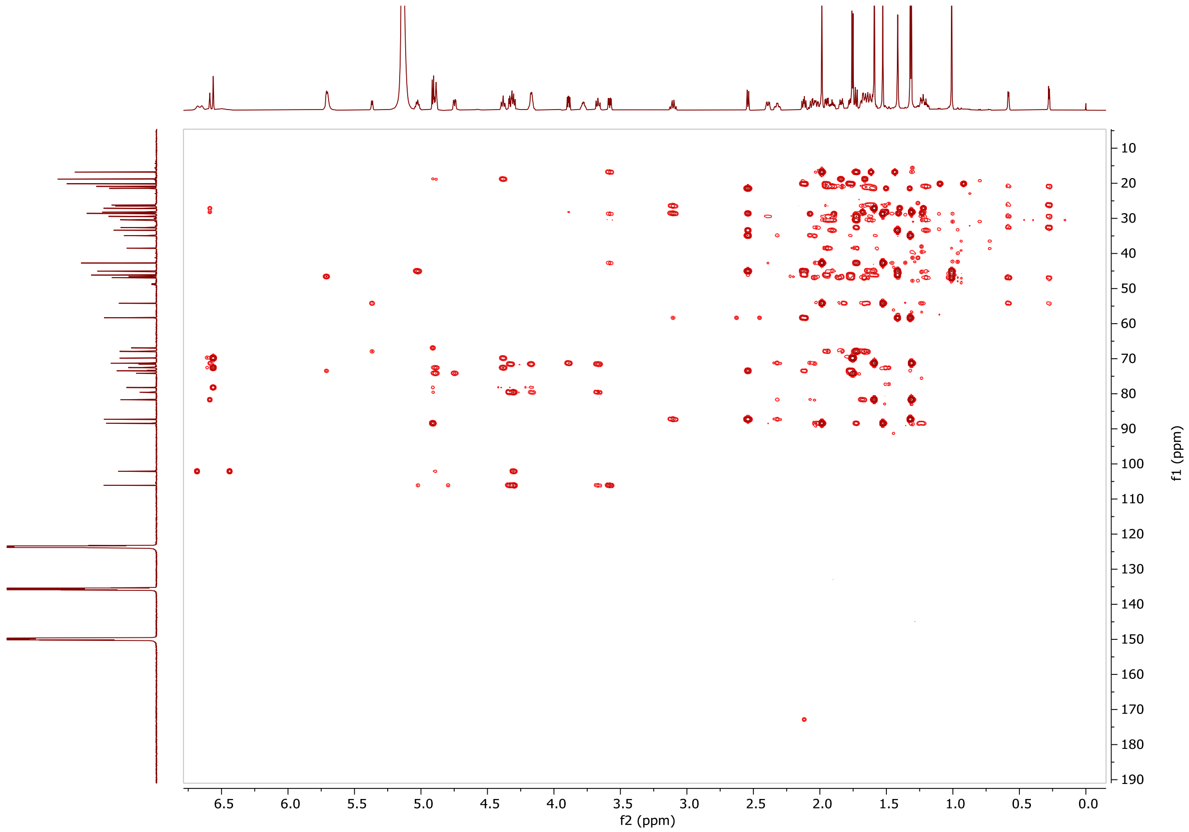

Figure S17: HMBC spectrum (700/175 MHz, pyridine-d_5) of compound **S2**.

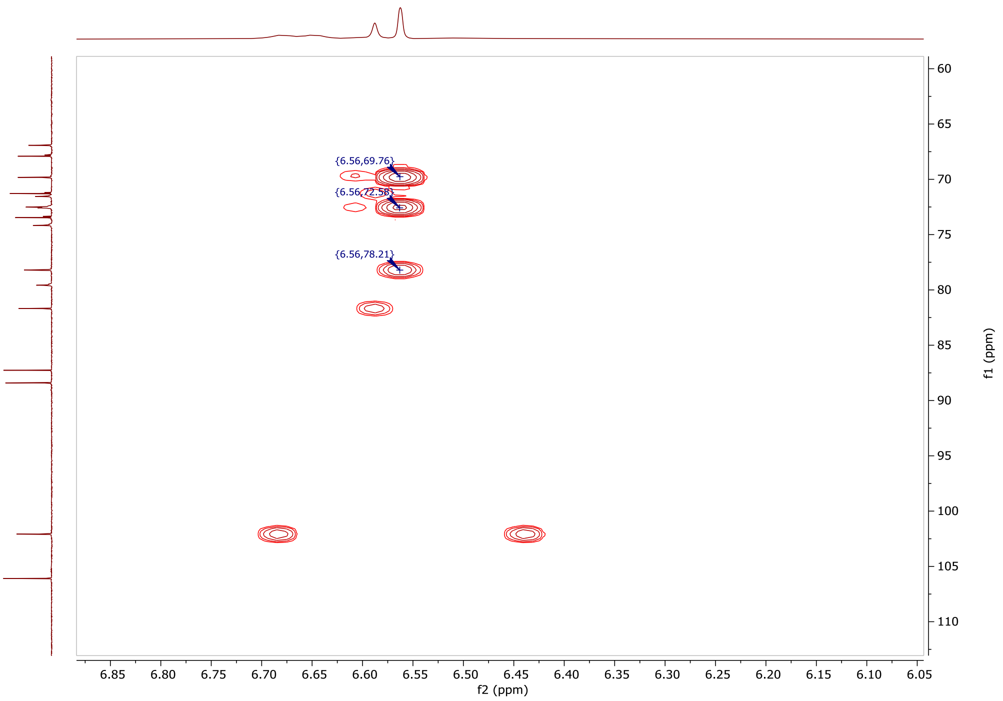

Figure S18: Expansion of the sugar region of the HMBC spectrum (700/175 MHz, pyridine-d_5) of compound **S2**.

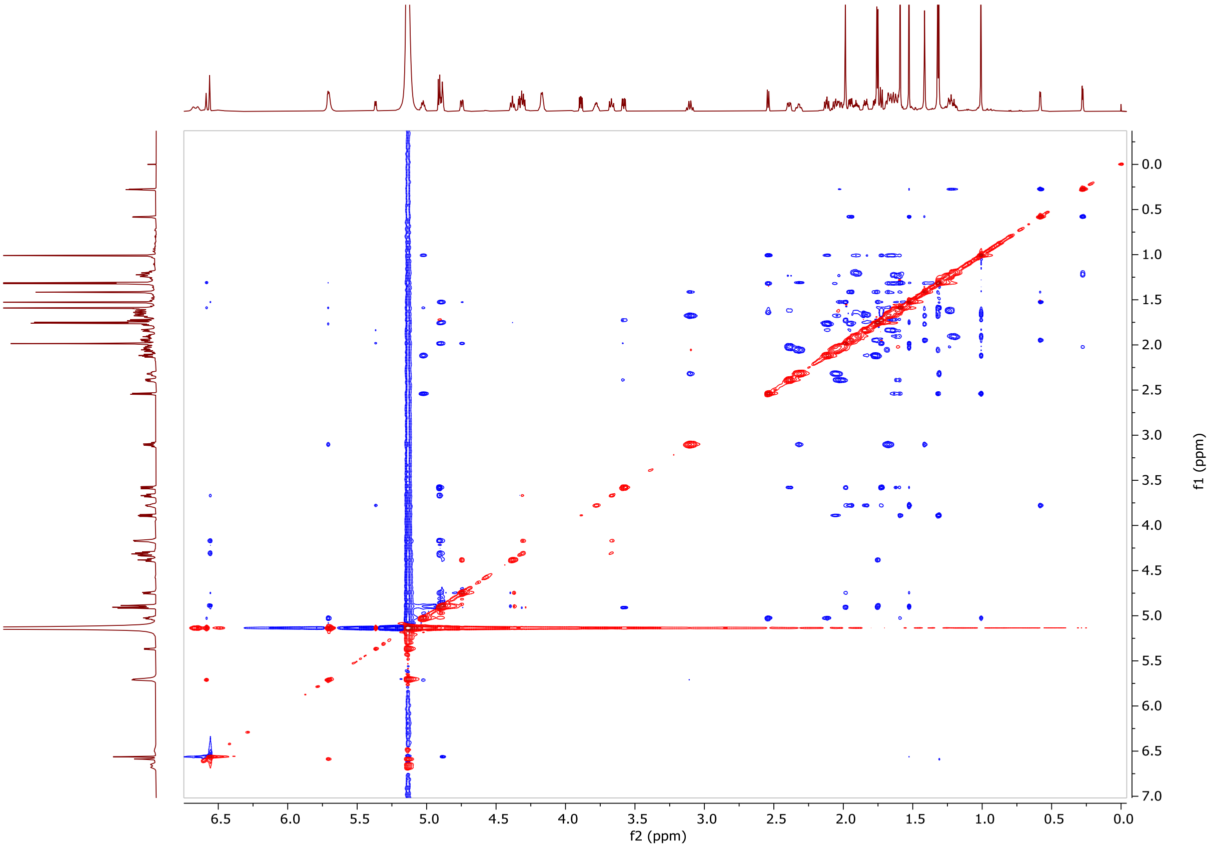

Figure S19: ROESY spectrum (700 MHz, pyridine-d_5) of compound **S2**.

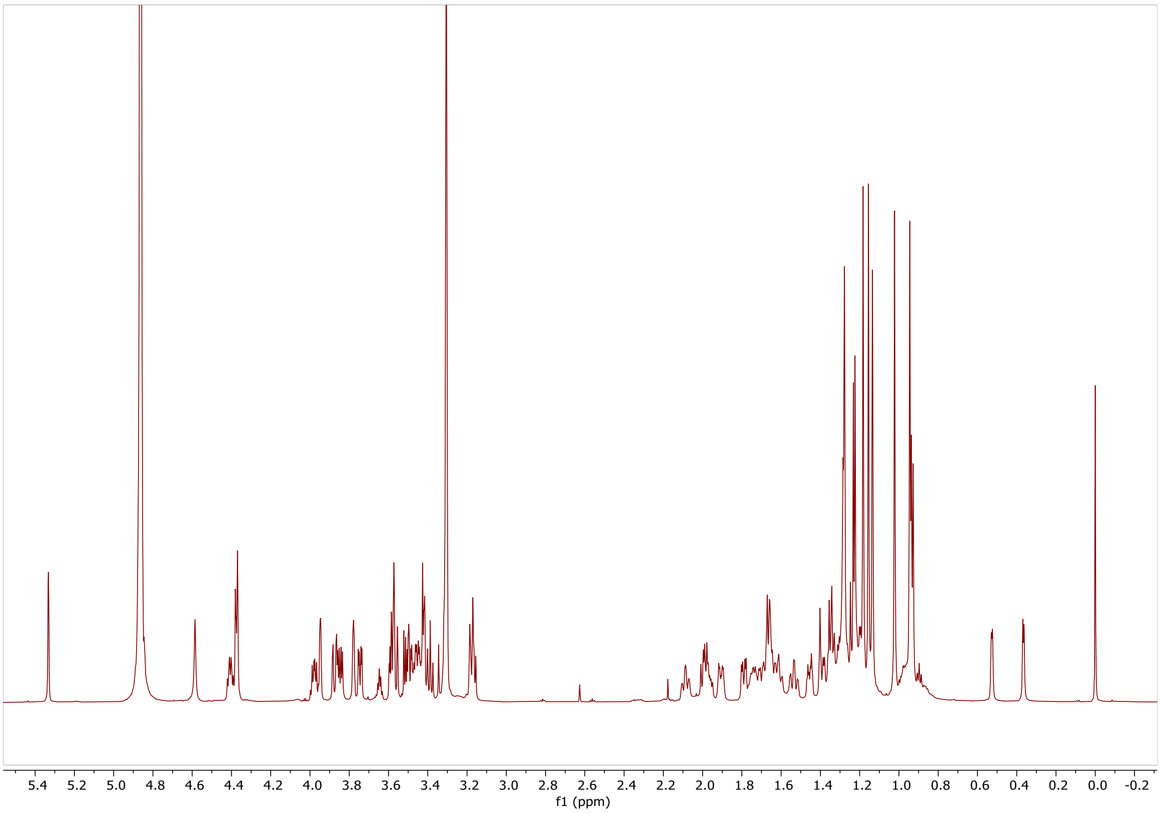

Figure S20: ^{1}H-NMR spectrum (700 MHz, methanol-*d₄*) of compound **S2**.

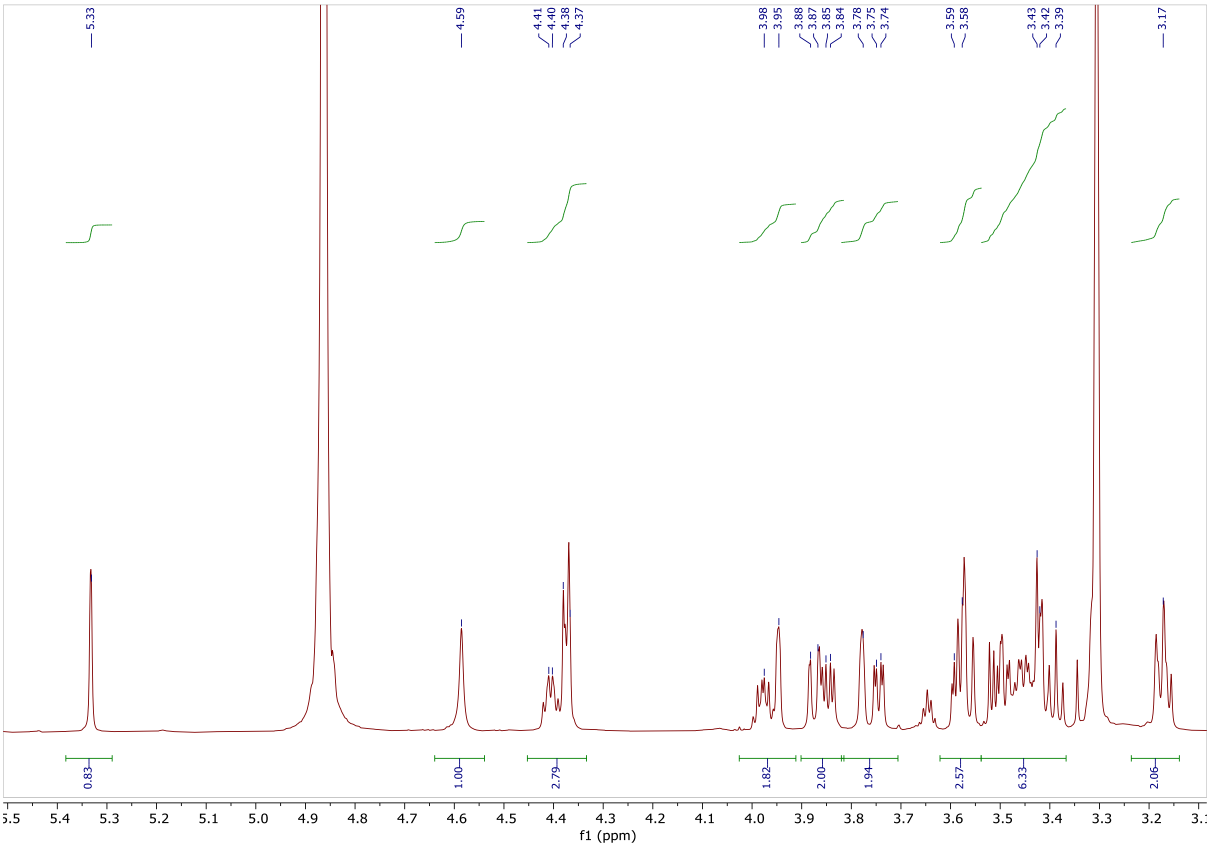

Figure S21: Expansion of the ^{1}H-NMR (700 MHz, methanol-*d₄*) spectrum of compound **S2.**

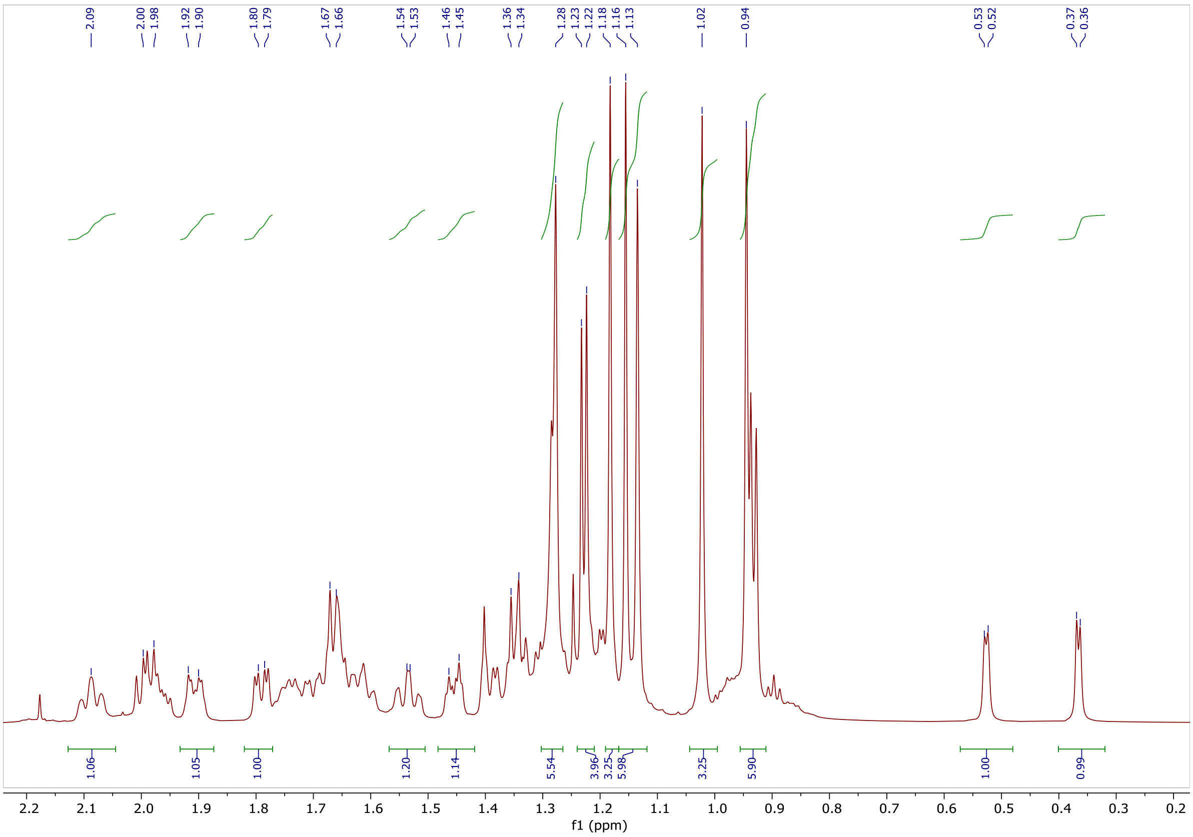

Figure S22: Expansion of the ^{1}H-NMR (700 MHz, methanol-d_4) spectrum of compound **S2.**

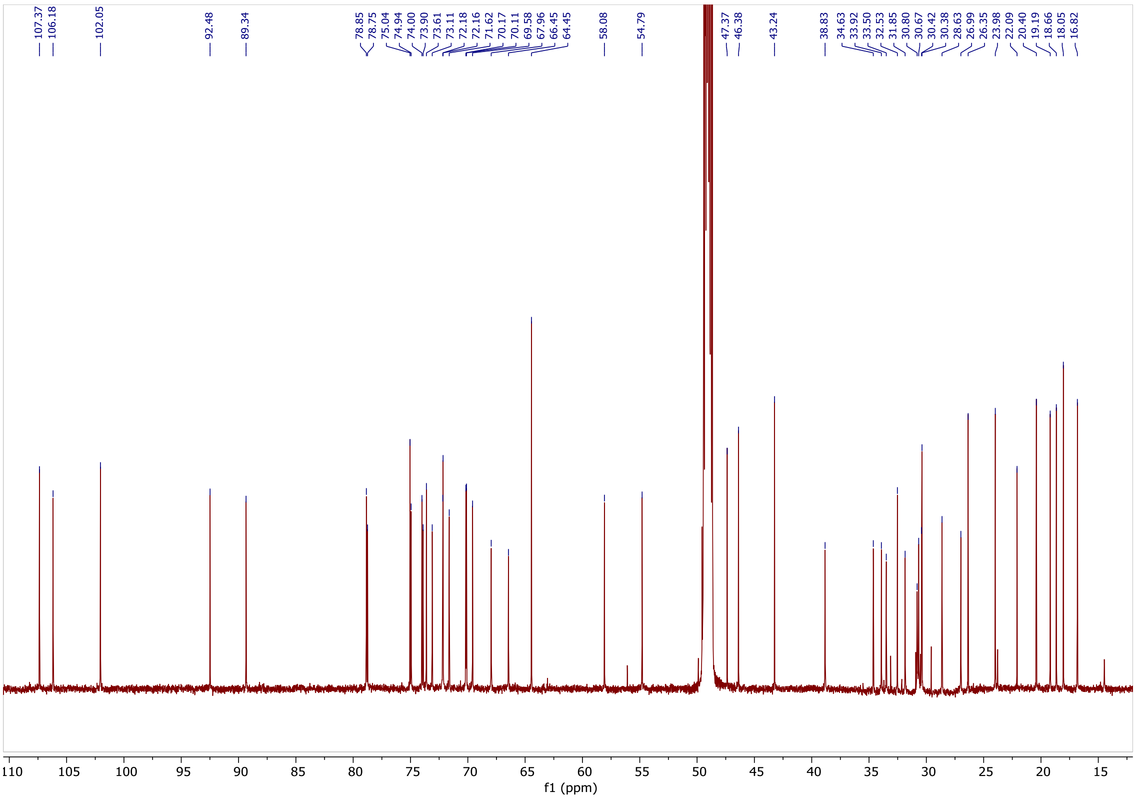

Figure S23: ^{13}C-NMR spectrum (175 MHz, methanol-d_4) of compound **S2**.

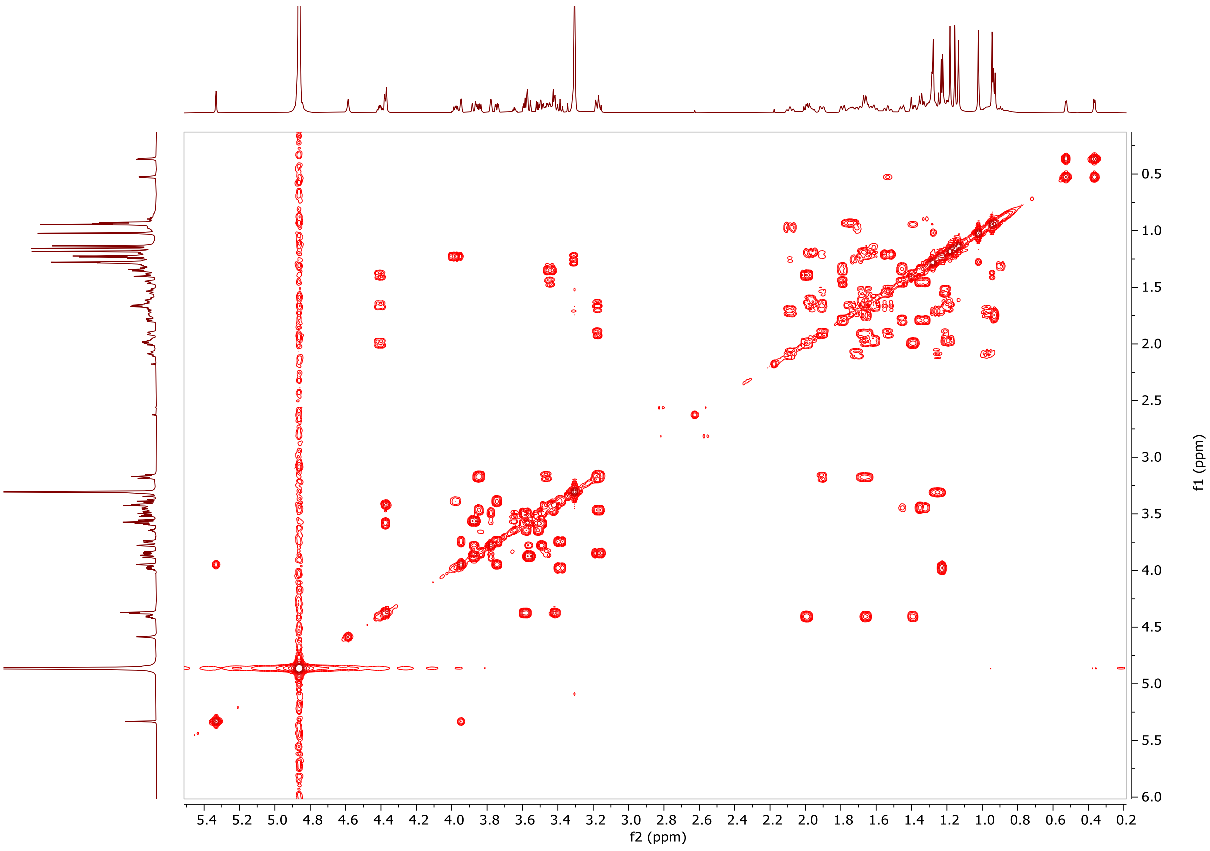

Figure S24: COSY spectrum (700 MHz, methanol-*d*$_4$) of compound **S2**.

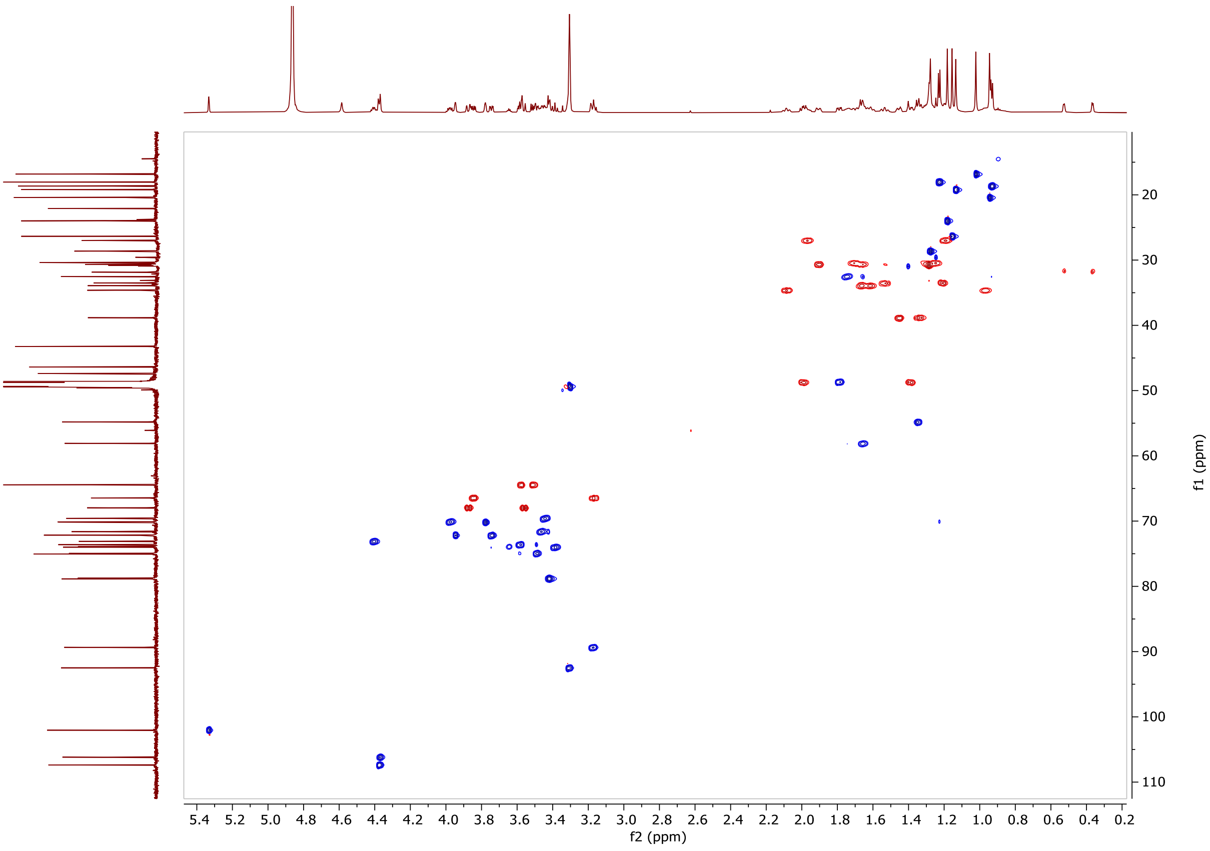

Figure S25: HSQC spectrum (700/175 MHz, methanol-*d*$_4$) of compound **S2**.

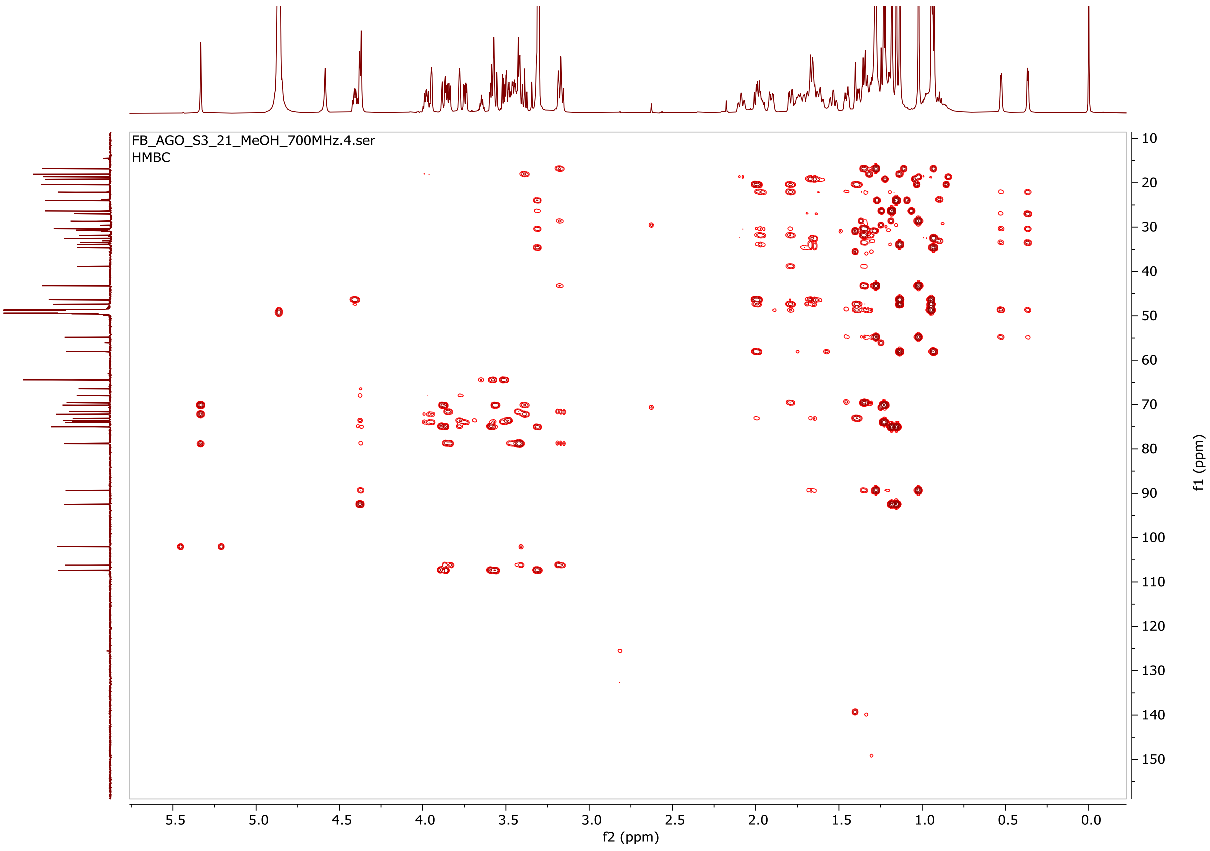

Figure S26: HMBC spectrum (700/175 MHz, methanol-d_4) of compound **S2**.

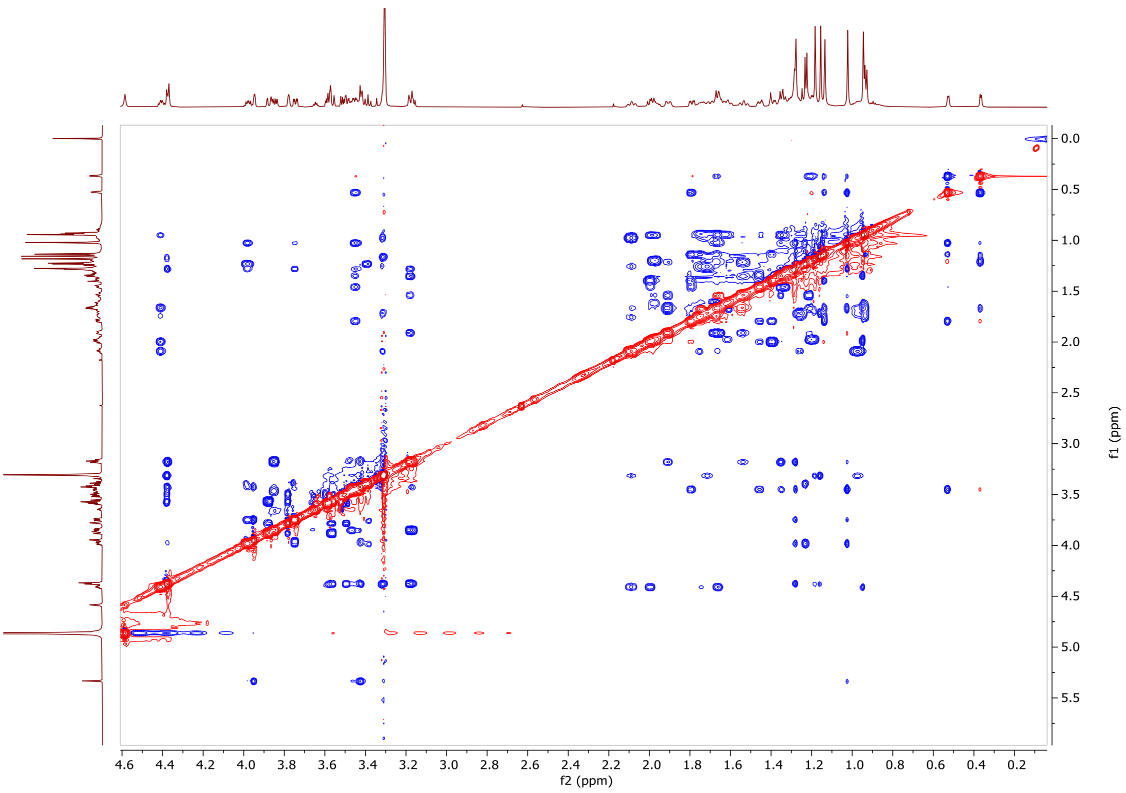

Figure S27: ROESY spectrum (700 MHz, pyridine-d_5) of compound **S2**.

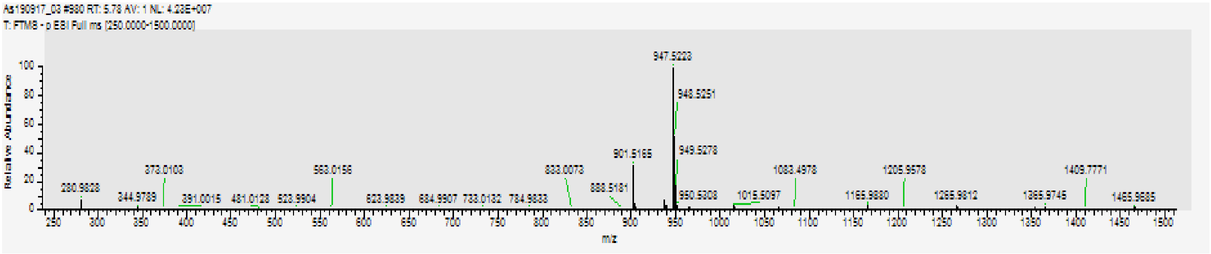

Figure S28: HRESIMS of S1 in negative mode

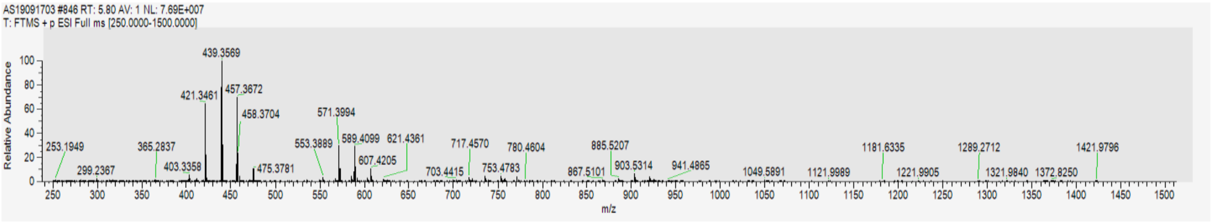

Figure S29: HRESIMS of S1 in positive mode

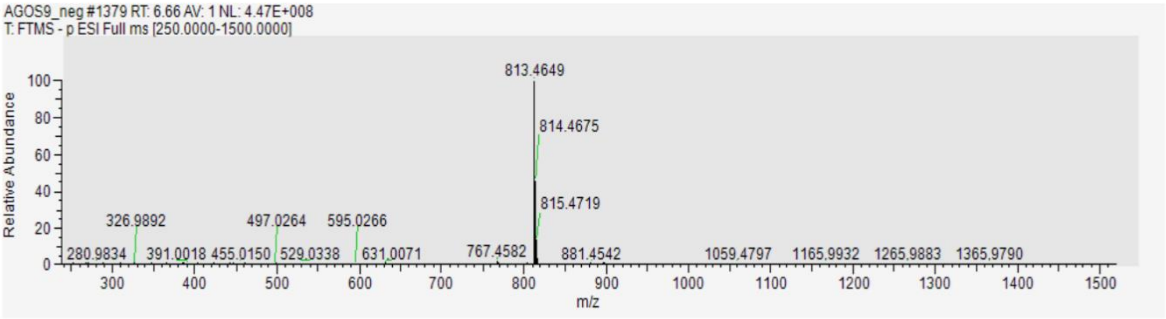

Figure S30: HRESIMS of S2 in negative mode

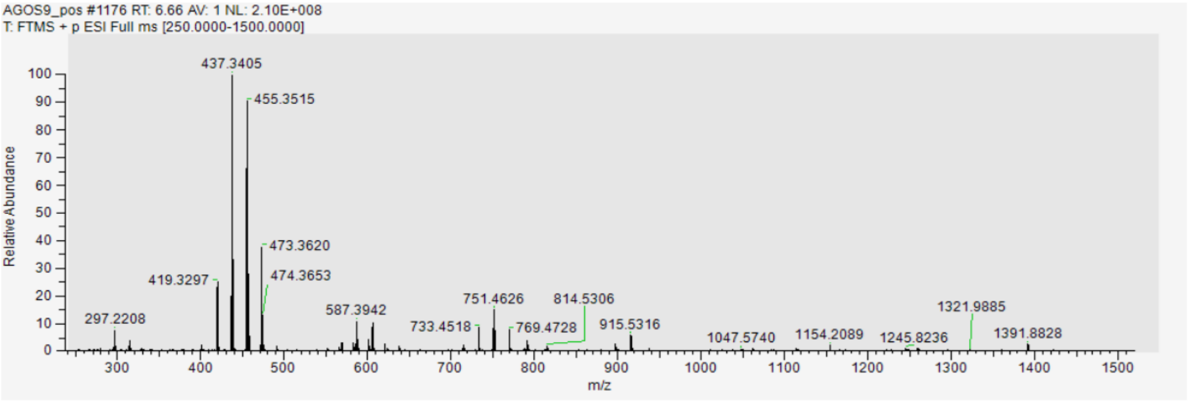

Figure S31: HRESIMS of S2 in positive mode

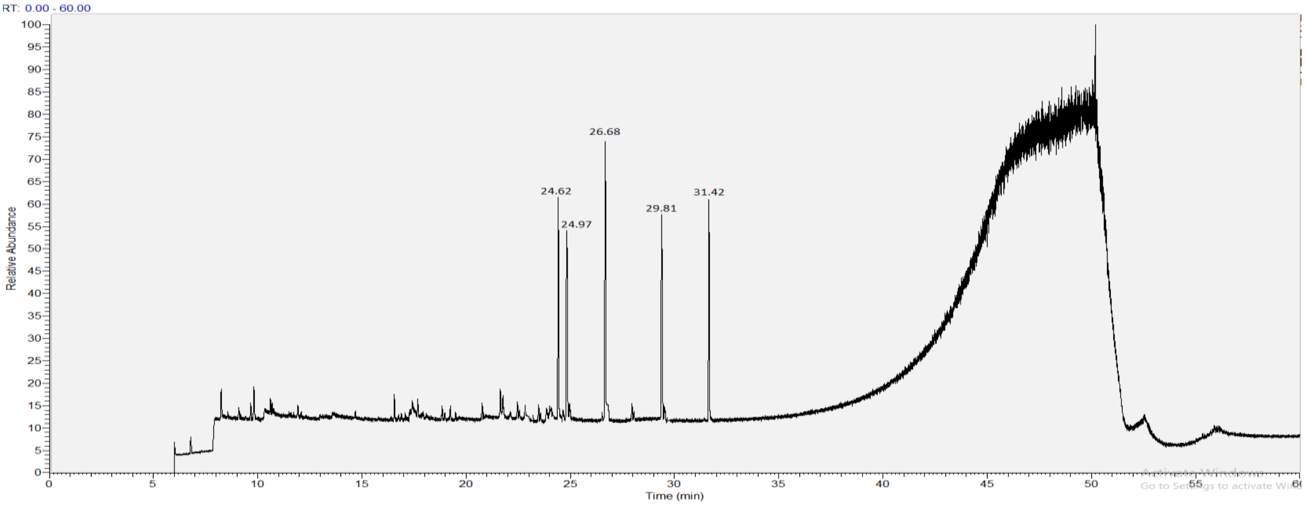

Figure S32: GC chromatogram of the monosaccharides of S1

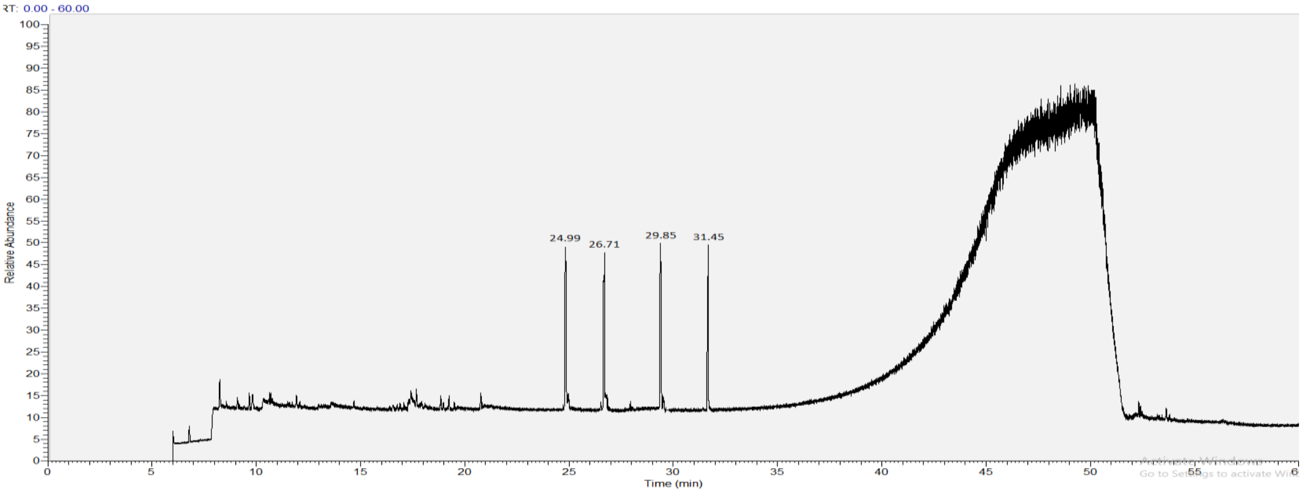

Figure S33: GC chromatogram of the monosaccharides of S2
